# Viscosity of Plasma as a Key Factor in Assessment of Extracellular Vesicles by Light Scattering

**DOI:** 10.3390/cells8091046

**Published:** 2019-09-06

**Authors:** Darja Božič, Simona Sitar, Ita Junkar, Roman Štukelj, Manca Pajnič, Ema Žagar, Veronika Kralj-Iglič, Ksenija Kogej

**Affiliations:** 1 Department of Chemistry and Biochemistry, University of Ljubljana, Faculty of Chemistry and Chemical Technology, SI-1000 Ljubljana, Slovenia; 2 Department of Polymer Chemistry and Technology, National Institute of Chemistry, SI-1001 Ljubljana, Slovenia; 3 Department of Surface Engineering and Optoelectronics, Josef Stefan Institute, SI-1000 Ljubljana, Slovenia; 4 Biomedical Research Group, University of Ljubljana, Faculty of Health Sciences, SI-1000 Ljubljana, Slovenia

**Keywords:** extracellular vesicles, exosomes, blood plasma, dynamic light scattering, static light scattering, shape characterization, viscosity of blood plasma

## Abstract

Extracellular vesicles (EVs) isolated from biological samples are a promising material for use in medicine and technology. However, the assessment methods that would yield repeatable concentrations, sizes and compositions of the harvested material are missing. A plausible model for the description of EV isolates has not been developed. Furthermore, the identity and genesis of EVs are still obscure and the relevant parameters have not yet been identified. The purpose of this work is to better understand the mechanisms taking place during harvesting of EVs, in particular the role of viscosity of EV suspension. The EVs were harvested from blood plasma by repeated centrifugation and washing of samples. Their size and shape were assessed by using a combination of static and dynamic light scattering. The average shape parameter of the assessed particles was found to be *ρ* ~ 1 (0.94–1.1 in exosome standards and 0.7–1.2 in blood plasma and EV isolates), pertaining to spherical shells (spherical vesicles). This study has estimated the value of the viscosity coefficient of the medium in blood plasma to be 1.2 mPa/s. It can be concluded that light scattering could be a plausible method for the assessment of EVs upon considering that EVs are a dynamic material with a transient identity.

## 1. Introduction

Extracellular vesicles (EVs) are membrane micro/nano-particles commonly found in biological samples including body fluids [1,2]. The concentration of EVs in ex-vivo samples of blood plasma was estimated at 10^10^ particles/mL [3], but may be increased as a result of various pathological conditions [4,5]. Despite intensive investigations of these membrane particles in the last few decades, numerous issues remain unresolved, an important one being the effect of pre-analytical handling of samples on the results [5,6,7,8,9,10,11,12]. The dynamics of a vesicle membrane and its contents in ex-vivo samples is poorly understood. A possibility of EV transformations due to the aggressive processes of isolation from body fluids should be taken into account. It was suggested [12] that EVs result from self-organization of the available physico-chemical components following their exposure to various external factors. Consequently, viewing EV isolates as composed of particles in the state as formed in the body is to some degree questionable. Furthermore, the analytical methods for EV determination often require specific markers. This implies ethical bias and a higher cost of analysis, and thereby prevents a better understanding of these diverse biological features. The development of methods for minimally invasive analysis of EVs in body fluids is therefore highly desirable. Here, the possibility to use static and dynamic light scattering (SLS and DLS, respectively) is explored to assess the size and shape of EVs in isolates as well as in blood plasma.

The techniques of static (SLS) and dynamic light scattering (DLS) and their combination are commonly used in research and industry of various nano-systems [13,14,15,16,17,18]. These techniques do not require destruction of the sample. A relatively small volume of the sample (less than a milliliter) is sufficient for analysis. The size range of those methods (1 nm–1 µm) [19,20] is consistent with the expected size of EVs. The size distributions can be obtained by DLS without any a priori assumption of the particles’ chemical nature or structure [21]. In well-defined samples (preferably monodisperse or with known polydispersity), the underlying model (based upon the movement of particles in a fluid continuum with a constant viscosity) is relevant to determine the hydrodynamic radius of the particles (*R*_h_) [16]. SLS, on the other side, gives another size parameter, the radius of gyration of particles (*R*_g_). Combining the DLS and SLS parameters (*R*_h_ and *R*_g_, respectively) yields the so-called shape parameter *ρ* (= *R*_g_/*R*_h_). The gross distribution of mass within the particles can be estimated by comparison of obtained *ρ* values with those of the particles with well-defined topology [17].

As for the shortcomings of previous studies using LS techniques for the EV assessment, the possibility of a comparison of the different studies is limited due to the diversity of analysed samples and the angular range of measurements (e.g., in works [14,15,22,23,24,25,26,27,28,29]). As EVs are a heterogeneous system, it was exposed that larger particles scatter more light than smaller ones, which underestimates the contribution of smaller particles [30]. Furthermore, the analysis of the measurements is performed within the assumptions that EVs are particles with well-defined identity (their number, size and shape are fixed during the measurement) and that they move in the ideal fluid with a constant viscosity.

Assuming the validity of the above, the blood plasma and isolates from blood plasma of 3 healthy donors were analysed. A commercial exosome standard was used as a reference. This study focused on the viscosity of the fluid in which EVs moved during the measurement. The size distributions of the particles in the samples were determined. The polydispersity effect on LS data was considered. The contribution of each population was extracted from the total LS intensity on the basis of the intensity weighted *R*_h_ distributions. The angular dependency of scattered light was then assessed for each population individually. For the comparison and complementation, other experimental techniques were used to measure the vesicles’ size: The asymmetric flow field flow fractionation coupled with the multi-angle light scattering detector (AF4/UV-MALS), the conventional flow cytometry (FCM), and the atomic force microscopy (AFM). As far as the authors’ knowledge is concerned, this is the first study reporting such complete LS analysis of EVs in blood plasma samples.

## 2. Materials and Methods

### 2.1. Materials

#### 2.1.1. Exosome Standards

A lyophilized exosome standard HBM-PEP-100/6 (sample designation ES; c.f. Table S1) of exosomes isolated and purified from human blood plasma of healthy donors was purchased from HansaBioMed LLC (Estonia) in the form of six 100 μg pellets supplied in separate vials. One pellet was suspended in 100 μL of deionized and filtered water (filter pore size 0.2 µm). The suspension was gently mixed, further diluted with 900 µL of filtered PBS buffer solution (phosphate buffered saline (PBS), pH 7.4. It was composed of 137 mM NaCl, 2.68 mM KCl, 10.14 mM Na_2_HPO_4_, and 1.84 mM KH_2_PO_4_) and gently mixed again. Two of the four samples were supplemented with 300 mM trehalose (D-(+)-trehalose dehydrate; *Saccharomices cerevisiae*; T9631, lot#SLBQ6030V, abbreviated as TRE) to the final TRE concentration of 25 mM. The final concentration of the exosome standard in these samples was 0.1 g/L. The LS measurements were performed immediately after sample preparation and the same samples were recovered for further measurements. A small volume of unfiltered sample was taken for the analysis by AFM and AF4/UV-MALS, while the remaining suspension was filtered through the 0.45 µm pore-sized and low protein-binding filter (Millex^®^-HV, PVDF) directly into the clean quartz cell for LS measurements. The filtered sample was analysed again with all the above mentioned methods. The samples were frozen in vapours of liquid nitrogen and stored at −80 °C (the required time to reach −80 °C was determined in a separate experiment by measuring the temperature in the vial during freezing with a thermocouple). After 2–3 weeks, the samples were thawed in a water bath at 25 °C (thawing time was approximately 15 min) and re-analysed at the same temperature (25 °C).

#### 2.1.2. Blood Plasma Samples

This study has been carried out in accordance with The Declaration of Helsinki and was approved by the Republic of Slovenia National Medical Ethics Committee, (approval number: 82/07/14). The donations of 3 healthy donors (authors of the article; 2 women, 1 man, 24–57 years old; sample designations HD1-3; c.f. Tables S2–S4) were established in the morning, ensuing at least 12-h after night fasting. A G21 type needles (length 70 mm, inside diameter 0.4 mm, Microlance, Becton Dickinson, USA) and 2.7 mL vacutainer tubes with trisodium citrate (BD Vacutainers, Becton Dickinson, USA) were used. The samples were then centrifuged for 30 min at 2000× *g* (centrifuge Centric 400/R, Tehtnica, Železniki, Slovenia) and 22 °C.

The supernatant (plasma) was collected down to 4–5 mm above the cell sediment. The plasma from the three tubes of each donor was pooled in a 15 mL Falcon tube and gently mixed by inverting. Each sample was divided into aliquots of 0.8 mL by preparing the parallel samples with and without TRE (25 mM) as described above. An aliquot of each sample to be analysed fresh was immediately transported to the laboratory of analysis on ice. At the same time, the others were frozen at −80 °C, transported on dry ice to the place of storage and then stored in the freezer at −80 °C for 7–10 days before being thawed in a water bath at 25 °C and re-analysed.

The EV isolates were prepared from two samples, HD1 and HD2, three from each. A slightly modified protocol based on ref. Diamant et al. (2002) [4], as published in Štukelj et al. (2017) [3], was used for isolation. Namely, 250 μL of plasma was put into a 1.5 mL tube and centrifuged for 5 min at 17,570× g (centrifuge Centric 200/R, Domel, Slovenia). A volume of 210 μL of the supernatant was carefully removed, with the attention not to disturb the separated fractions, and then 210 μL of the filtered PBS-citrate buffer was added in order to re-suspend the pellet. The samples were centrifuged again for 5 min at 17,570× *g*. 210 μL of the supernatant was discharged (pipetting carefully from the top again), the remaining part (40 μL) was added to 60 μL of the fresh buffer. The pellet was re-suspended by pipetting up and down and 60 s slow-speed mixing on vortex. Further, 25 μL of the prepared sample was used for FCM, while the remaining 75 μL was diluted with 425 μL of the buffer. This sample was divided into aliquots for AFM, AF4/UV-MALS and the DLS/SLS measurements. The aliquots were transported to the place of analysis on ice and analysed on the same day.

#### 2.1.3. Blood Plasma for the Ultracentrifugation Experiments and Viscosity Determination

The blood plasma for the viscosity examination was provided by the Blood Transfusion Centre of Slovenia. The blood sample was collected by the standard procedure and in accordance with general standards for blood products preparation for transfusion purposes. The collecting bag was COMPOFLOW^®^ 4F T&B 63 mL CPD/100 SAGM–RCC–PDS-V, serial no. 41LD11FA00, Fresenius Kabi AG, Czech Republic). Overall, 453 mL of blood was donated without any complications (complete dose, collection time 4 min 34 s, scale and mixer device Hemoflow 06393). The 14 min centrifugation at 22 °C and 3540 rpm (Cryofuge 6000i, rotor 6606, Thermo Scientific, ZDA) for the cell separation was performed after 3 h 20 min of incubation of the blood at room temperature. The separation of plasma from the cell fractions was done automatically (CompoMat G5 and CompoMaster Net G5 System, program of separation: TB Buffy Coat). The obtained plasma (260 mL) was rejected and excluded from further medical utilisation because of the imperfect cell elimination. The sample was kept at room temperature (22 °C) approximately 20 h before it was handed over to the authors for the research purposes. The majority of plasma was dispensed into the 15 mL aliquots, frozen and stored at −80 °C, while the first experiment was started using a fresh sample right after reception. For the second experiment, the fresh plasma sample was stored in the fridge (at 8–10 °C) for two days. For the third experiment, a frozen sample was rapidly thawed at 37 °C. Fast thawing is recommended as crystal formation and cell destruction is supposed to be reduced. The plasma was put into the 1.5 mL tubes and centrifuged for 30 min at 5000× *g* at room temperature to eliminate all the cellular debris and possible other contaminants. Then, 1 mL of the supernatant from each tube was collected and pooled to get a clear starting sample. Further, 1.2 mL aliquots were put to the ultracentrifuge. To test the effect of viscosity on the ultracentrifugation efficiency, two samples were diluted with the PBS buffer in plasma to buffer ratios 1:1 and 1:2. Ultracentrifugation at 100,000× *g* (38,000 RPM, ultracentrifuge Beckman L8-70M, rotor Ti50.2; (Beckman Coulter, USA) was sustained for 8 h at 8–10 °C.

#### 2.1.4. Human Serum Albumin

(HAS; Sigma Aldrich, USA, A9511-1G, lot. #SLBL5290V) solution was prepared by dissolving the protein in PBS at the final concentration of 70 mg/mL. The solution was filtered through a low protein binding filter with a pore size of 0.2 µm.

### 2.2. Methods

#### 2.2.1. Static and Dynamic Light Scattering Measurements

The DLS and SLS measurements were used to determine the *R*_h_ and *R*_g_ of particles in the samples. All LS measurements were conducted with the 3D-DLS-SLS cross-correlation spectrometer from LS Instruments GmbH (Fribourg, Switzerland). As the light source, a 20 mV He-Ne laser (Uniphase JDL 1145P, Thorlabs, Newton, NJ, USA) with a wavelength *λ*_0_ = 632.8 nm was used. All the details regarding the instrumentation were reported previously [31,32]. The samples were equilibrated in the decalin bath at 25 °C for 15 min prior to performing the measurement.

A very careful mathematical analysis of the LS measurements was performed (for details, see Section S1.1 in SM). The correlation functions and the integral time averaged intensities *I*(*θ*) ≡ *I*(*q*) at an angle *θ* or the corresponding scattering vector *q* (=(4π*n*_0_/*λ*_0_)*sin*(*θ*/2)), where *n*_0_ is the refractive index of the medium) were recorded simultaneously. For *n*_0_ the value of water (*n*_0_ = 1.33) was used. The intensities were normalized with respect to the Rayleigh ratio of toluene (*R*_T_) and converted into the absolute intensity units (cm^−1^). Together with the absolute LS intensity of the samples (*R*), the absolute LS intensity of the solvent (*R*_0_) was measured, which enabled the calculation of the excess LS intensity of the samples, expressed as Δ*R* (= *R* − *R*_0_). For the purpose of measuring *R*_0_, water was chosen as the solvent. All the measurements were performed in the angular range between 30° and 150° with a step of 10° or 20°. The constant intensity of light scattered at an angle of 90° was used as the criterion that the solution was properly equilibrated. The measurements of the angular dependency of the scattered light intensity enable the determination of the form factor (*P*(*q*)) of colloidal particles from *P*(*q*)=*I*(*q*)/*I*(0), where *I*(0) is the LS intensity extrapolated to *θ* (or *q*) = 0. Five intensity correlation functions were collected at each angle and averaged. Each curve was analysed independently and compared with the averaged curve to ensure accuracy of the mathematical solution.

The detailed methodological aspects of the SLS and DLS can be found elsewhere [17,33]. The additional experimental information regarding the LS measurements together with the procedures used for the data treatment in this paper is described in detail in SM. A brief explanation is given below.

In DLS, the fluctuations of the intensity of scattered light are presented as the correlation function of the scattered light intensity, *G*_2_(*t*), where *t* is the time on the relaxation time axis. In order to determine the diffusion coefficient, *D*, of particles, the *G*_2_(*t*) function is converted into the correlation function of the scattered electric field, *g*_1_(*t*), by using the Siegert’s relationship [17,33]. The relaxation time, *τ*, of the *g*_1_(*t*) function is related to the relaxation rate, *Γ* = *τ*^−1^, and the translational diffusion coefficient, *D*, of particles by the relationship:

(1) $$\left|g_{1}\left(t\right)\right|=e^{-t/\tau }=e^{-\Gamma t}=e^{-Dq^{2}t}$$

The value of *R*_h_ is obtained from *D* via the Stokes-Einstein equation (Equation (2)) by assuming the spherical shape of the particles.

(2) $$R_{h}=\frac{kT}{6\pi \eta D}$$

In Equation (2), *k* is the Boltzmann constant, *T* is the absolute temperature, and *η* is the viscosity of the solvent/medium, in which the particles diffuse. The correct viscosity for accurate *R*_h_ determination is one of the focuses of this study.

Equation (1) (the mono-exponential form) is strictly valid for monodisperse spherical particles. For polydisperse samples, several exponents appear in this expression and a multi-exponential fit of *g*_1_(*t*) has to be performed. The multi-exponential fit was achieved by the inverse Laplace transformation program CONTIN, resulting in the distribution of the relaxation times of species in solution. The distributions over *τ* were converted into size (*R*_h_) distributions by means of the relationship *τ*^−1^ = *Dq*^2^ and Equation (2).

The essential scattering properties of EVs were determined based on the analysis of the exosome standard. To assess if it is possible to analyse the EVs by the batch DLS/SLS in the complex sample of a human blood plasma, the blood plasma of three healthy donors (HD1–3; see Tables S2–S4 in SM) was analysed along with the EV isolates prepared from them, and the results were compared with those of the exosome standards (ES1–4; Table S1, SM). Attention was devoted also to the effect of filtering and freezing on the samples as such and on the DLS/SLS results, as those procedures are routinely used in EV handling, but also represent the potential sample modifiers. The role of trehalose as a possible cryo-protectant and aggregation preventer [34] was also tested. The reader is referred to SM (Table S1, Section S2) for details on these results and additional discussion.

For the herein studied samples, all the size distributions were multimodal, exhibiting well-resolved 2–3 peaks. An example of such *R*_h_ distribution is reported in Figure 2 and Figure S1 together with the method of calculating the contributions of the peak referring to the population of EVs (c.f. Figure S1a) to the total LS intensity. It has to be stressed that the time-averaged intensities of the individual peaks (individual particle populations) were extracted from the total LS intensity on the basis of the intensity weighted distributions of *R*_h_ and were then analysed separately for the angular dependency in order to determine the mean *R*_g_ (an SLS data) value of a particular particle population. The detailed procedure of this approach has been outlined in references [35,36,37] and is demonstrated for one of the samples studied here in SM (see Figure S1a and Equations S4 and S5 in Section 1.1.2, SM). The relevant equations for the evaluation of *R*_g_ from the form factor *P*(*q*) of individual populations are also reported in SM (Equations S3a–e; the derivation of the *P*(*q*) for vesicles-Equations S3b and S3c-is given in Section S1.1.3.1). As this DLS and SLS analysis is based on the LS measurements performed on the whole sample and the contributions of individual populations are extracted and analysed afterwards, the expression batch DLS/SLS for this approach has been used.

The particle size characteristics obtained by the SLS and DLS, i.e., *R*_g_ and the zero angle *R*_h_ values (notation *R*_h,0_), respectively, were combined to give the shape parameter *ρ* (= *R*_g_/*R*_h,0_). The *ρ*-ratio provides an important indication of the scattering particle topology (shape and mass distribution). For the present study, the following *ρ*-values are noteworthy: *ρ* of a hard (homogeneous) sphere is *ρ* = 0.775 and that of a hollow sphere, which is the most appropriate shape approximation for vesicles, is *ρ* = 1 [17].

#### 2.2.2. Viscosity and Density Measurements

Kinematic viscosity (*η*) was measured with a micro Ubbelohde viscometer (type and capillary no. 537 10/I, apparatus no. 1070016; SI Analytics GmbH, Mainz, Germany) and an automatic flow time measuring system ViscoSystem^®^ AVS 370 (SI Analytics GmbH, Mainz, Germany). The density (*d*), which is needed for calculating dynamic viscosity, *η*, was measured with a vibrating tube densitometer (DMA5000, Anton Paar GmbH, Germany). All details regarding *η* and *d* measurements were reported previously [38]. The cumulative error in *η* determination was estimated to be ±1%.

#### 2.2.3. Asymmetric Flow Field Flow Fractionation (AF4/UV-MALS)

The AF4 was performed on Eclipse 3+ system (Wyatt Technology Europe, Germany) connected to the isocratic pump, on-line vacuum degasser and auto-sampler (Agilent Technologies 1260 series, USA). The samples were separated in a trapezoidal-shaped channel, with a tip-to-tip length of 152 nm and an initial channel breadth of 21 mm that decreased to final 3 mm, equipped with the 350 µm spacer and 10 kDa regenerated cellulose (RC) membrane. The fractionated particles were detected with an on-line UV detector operating at 280 nm (Agilent Technologies, USA) and a multi-angle light scattering (MALS) detector (DAWN HELEOS, Wyatt Technology, Goleta, CA, USA) operating at a wavelength of 658 nm, calibrated using toluene and normalized with bovine serum albumin protein as an isotropic scatterer standard. As a running eluent, PBS buffer (phosphate buffered saline, pH 7.4, composed of 137 mM NaCl, 2.68 mM KCl, 10.14 mM Na_2_HPO_4_, and 1.84 mM KH_2_PO_4_) supplemented with 0.02% w/v sodium azide as a bactericide was used. The eluent was filtered through a Nylon 66 membrane with a pore-size of 0.45 µm (Supelco Analytical, USA). Between the HPLC pump and the AF4 channel, an additional filter with a pore-size of 0.1 µm was placed (PEEK Inline Filter Holder, Wyatt Technology, Goleta, CA, USA).

The samples were injected in focus mode using the focus flow of 1.5 mL/min and the injection flow of 0.2 mL/min over 5 min. After injection, the samples were focused for additional 7 min. Under such focusing conditions, the exosomes do not undergo deformation or aggregation, nor interact with the membrane [39]. The elution of the samples was performed at the detector flow rate of 1.0 mL/min using two linear cross-flow gradients, 0.55 mL/min^2^ (cross-flow decreasing from 3.0 mL/min to 0.25 mL/min in 5 min) and 0.0027 mL/min^2^ (cross-flow decreasing from 0.25 mL/min to 0.09 mL/min in 60 min). The lowest cross-flow limit of the instrument was 0.09 mL/min. Once this limit was reached, the cross-flow was turned off. The last step was washing the channel for 10 min in elution mode without using any cross-flow.

The ASTRA 5.3.4.20 software from Wyatt Technology (Santa Barbara, CA, USA.) was utilized to analyse the data. The size of the exosomes was expressed as the radius of gyration (*R*_g_). This is determined by MALS without any need to know the solute concentration and/or sample refractive index increment. The *R*_g_ values of the fractionated exosomes were calculated using the data from 15 angles from the MALS detector, applying the Debye 2nd order approximation. The method for exosome separation and size characterization was developed previously [39]. For some additional support of the method, see references [40,41,42].

#### 2.2.4. Flow Cytometry (FCM)

The particle count was performed using 25 µL of samples by MACS Quant flow cytometer (Miltenyi Biotec GmbH, Bergisch-G Ladbach, Germany) with 405 nm, 488 nm, and 640 nm air-cooled lasers. Further details of FCM are explained in SM.

#### 2.2.5. Atomic Force Microscopy (AFM)

The EV isolates were immobilized to the anatomically flat freshly cleaved mica. The EVs were incubated with mica surface for 20 min. After incubation, the unattached EVs were gently rinsed with water and the samples were analysed by AFM. The analysis was conducted by the AFM Solver PRO (NT-MTD, Moscow, Russia) in tapping mode in air at room temperature. The samples were scanned with a standard Si cantilever (NT-MDT, NSG10, Moscow, Russia) with a force constant from 3.1–37.6 N/m at a resonant frequency of 155 kHz (tip curvature radius was 10 nm).

## 3. Results

**Figure 1:**
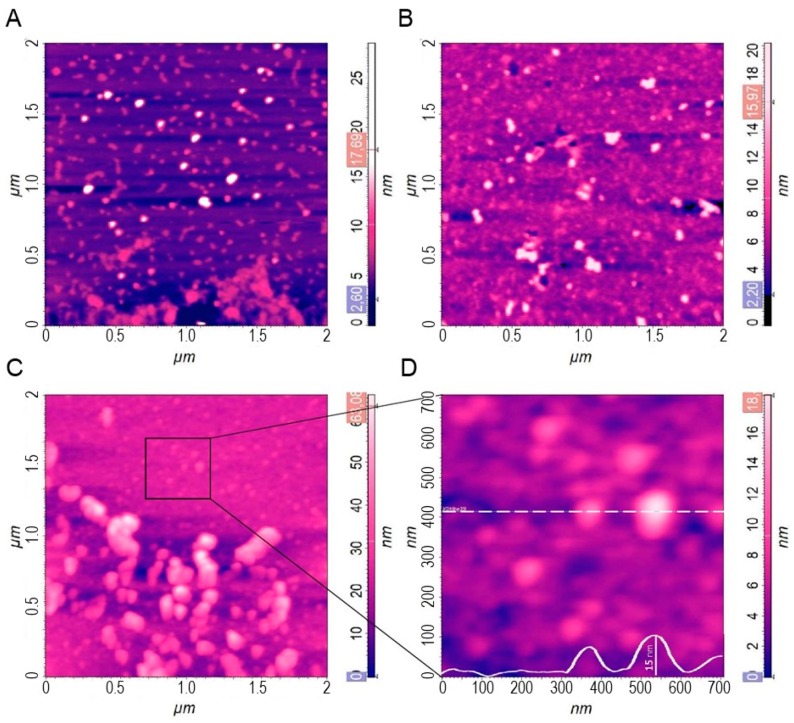
Atomic force microscopy (AFM) micrographs of (**A**) an unfiltered, (**B**) filtered exosome standard, (**C**) an extracellular vesicle (EV) isolate from a blood plasma sample, and (**D**) magnification of the area indicated in C.

Some AFM micrographs of representative samples are shown in Figure 1. The background of the EV isolate (Figure 1C) is much stronger than the one of the exosome standard (Figure 1A,B) due to the higher amount of proteins in isolates. The large particles in EV isolate (Figure 1C) were approximately 100–200 nm in diameter. However, the individual particles in the background (Figure 1D) were of similar sizes as the ones in ES (roughly 50–100 nm). The AFM micrographs thus clearly show that the samples are composed of larger particles and of considerably smaller ones (below 50 nm in diameter), constituting the background medium. The larger particles were analyzed by LS.

### 3.1. LS Size Characterization of the Exosome Standard

**Figure 2:**
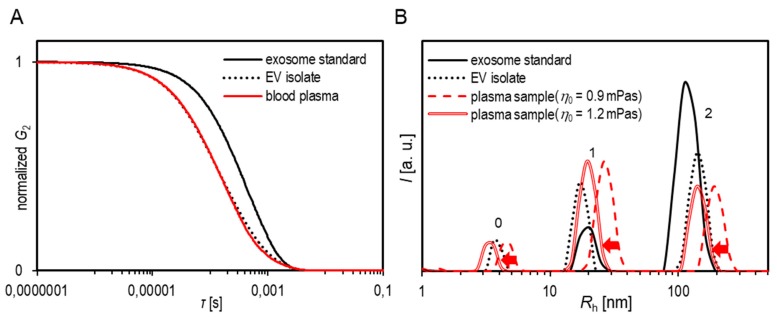
(**A**) The measured correlation functions *G*_2_(*t*) and (**B**) the calculated size distributions of particles in an unfiltered exosome standard (solid black line), a fresh plasma sample (dashed red line) and the corresponding EV isolate from the same sample (dotted black line). In calculation of *R*_h_ (Equation (2)) for the exosome standard, the viscosity coefficient of water (*η*_0_ = 0.9 mPa/s) was used, while for the plasma sample both, water (dashed red line) and the estimated viscosity of the plasma medium (*η* = 1.2 mPa/s; double red line; for details see text below) were taken into account. The arrows depict the shift of peak positions in the plasma sample after the viscosity correction.

In agreement with the AFM micrographs, the size-distributions of particles in the exosome standard as obtained by batch DLS were bimodal (or trimodal in some thawed samples). The polymodality is demonstrated for an exosome standard in the *lng*_1_(t) versus *t* plot in Figure S1B. As exosome suspensions were very dilute (see Materials and Methods), the viscosity of water *η*_0_ (= 0.90 mPa/s at 25 °C) was used to calculate *R*_h_ from Equation (2). A representative *R*_h_ distribution is shown in Figure 2 (c.f. the solid black line). The peaks with *R*_h_ < 10 nm (labelled 0) were assigned to free proteins and other small particles, while two peaks (labelled 1 and 2) were generally found in the size range expected for EVs. The mean *R*_h_ values of peaks labelled 1 obtained at *θ* = 90° (see *R*_h,90_ values reported in Table S1, SM) were between 10 and 30 nm (diameter between 20 and 60 nm) and those of peaks labelled 2 were between 100 and 150 nm (diameter between 200 and 300 nm). The corresponding populations are from here on termed *population 1* (small vesicles) and *population 2* (large vesicles). The *R*_h_ values of population 1 were largely independent of the angle of observation and therefore the value at *θ* = 90° (*R*_h,90_) or the average *R*_h_ (*R*_h,aver_) was evaluated and used in the calculation of *ρ* (see *ρ* values in Tables S2–S4, SM). On the other hand, the *R*_h_ values of population 2 displayed considerable angular dependence (c.f. Figure S5a). The extrapolation to *θ* = 0 with the purpose to determine the *R*_h,0_ values was done by linear regression, as shown in Figure S5a. The *R*_h,0_ values are reported in Table S1 (SM).

**Figure 3:**
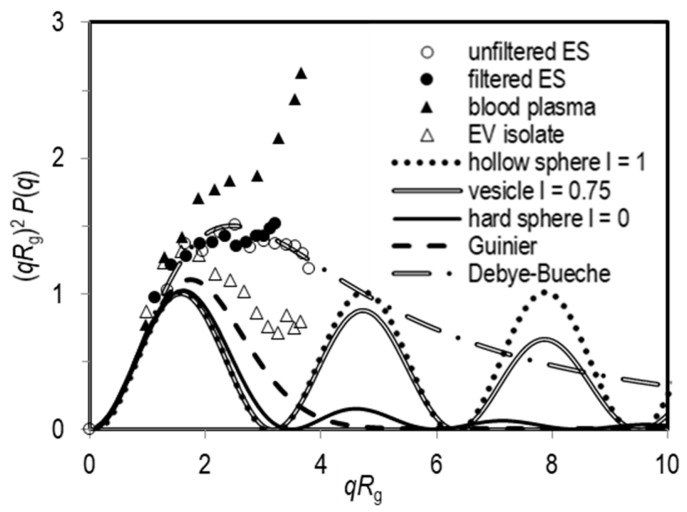
The intensity of scattered light measured by static light scattering (SLS) and represented as the Kratky plot (i.e., (*qR*_g_)^2^*P*(*q*) versus *qR*_g_) for the population 2 (large particles; see text) in the exosome standard ES (open circles: unfiltered; full circles: filtered), a plasma sample (full triangles) and the corresponding EV isolate from the same sample (open triangles). The lines show the calculated (*qR*_g_)^2^*P*(*q*) versus *qR*_g_ dependencies for some selected shapes (for details see Supplementary Material).

In the SLS analysis of population 1, the intensity of scattered light varied only slightly with the angle, meaning that *R*_g_ for this population is poorly defined. This is expected, as it turned out that these particles are mostly smaller than 1/20 of the He-Ne laser wavelength. Meanwhile, the angular dependency of the scattered light intensity was generally well-expressed for population 2, enabling extraction of the respective *R*_g_. An example of the angular dependency of the LS intensity of population 2 for the unfiltered and filtered exosome standard is shown in Figure S5B (as (*R* − *R*_0_)^−1^ = *f*(*q*^2^) plot) and in Figure 3 in the form of a Kratky plot (i.e., (*qR*_g_)^2^*P*(*q*) versus *qR*_g_). It can be seen that the experimental data for the exosome standard analysed by the batch DLS/SLS approach agreed best with the Debye-Bueche scattering function (Equation S3d, SM), which is valid for spherical microgel-like particles [43,44]. A deviation of the experimental points from the theoretical form factors of various spherical shapes (vesicle, hollow sphere or hard sphere) is clearly seen (Figure 3). These deviations are expected for polydisperse particles, as demonstrated experimentally and computationally in the literature [45,46,47]. The differences between the experimental and theoretical results increase with increasing polydispersity (this can be appreciated even more evidently for EVs in the blood plasma sample in Figure 3; see discussion below). The eventual aggregates of particles can contribute to this feature [45,47]. As reported in the literature on egg yolk phosphatidylcholine vesicles [39], the characteristic oscillations of form factors for spherical shapes (see the dotted, double and full lines in Figure 3) can be lost because of this polydispersity effect.

**Figure 4:**
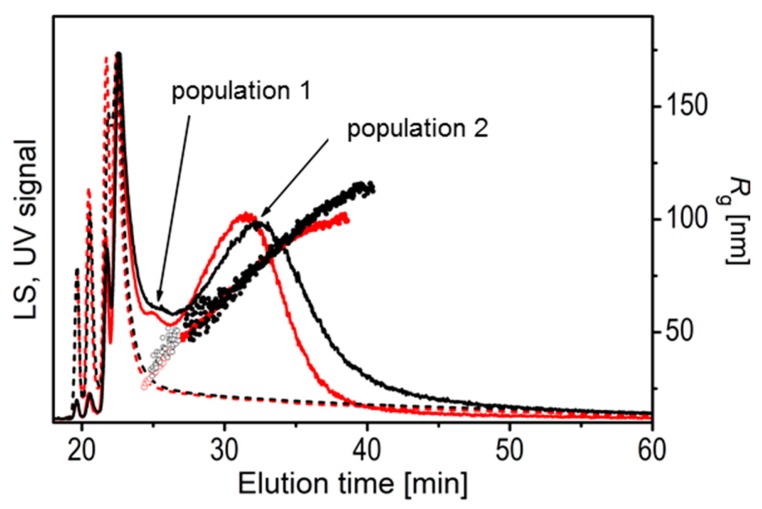
The AF4/UV-MALS fractograms of unfiltered (black) and filtered (red) exosome standards (ES) together with radius of gyration *R*_g_ as a function of time for smaller (open circles) and larger (full circles) vesicle populations. The solid lines represent the LS detector responses at 90° angle, while the dashed curves represent the UV detector responses.

The unfiltered and filtered ES sample (ES1) was analysed also by AF4/UV-MALS (Figure 4). Similar to batch DLS, AF4/UV-MALS revealed 2–3 major populations in the samples, differing in the particle size. The fractograms recorded by the UV detector show high UV intensity peaks at shorter elution times (below 25 min), which are characteristic of proteins. On the other hand, low intensity UV response and high intensity broad LS response at longer elution times (centred at around 32 min) indicate the presence of vesicles; the larger ones represent the population 2, while the smaller ones, which can be seen as a poorly resolved peak at ~25 min, centred between the proteins and large vesicles (Figure 4), can be linked to the population 1. The AF4/UV-MALS results are thus in line with the DLS results.

The *R*_h_ and *R*_g_ values of the two exosome populations as determined by batch DLS/SLS and AF4/UV-MALS are reported in Table 1 for one of the exosome standards (more DLS/SLS results can be found in Table S1). The average *R*_g_ values as determined by AF4/UV-MALS were ~100 nm for large vesicles (population 2) and ~40 nm for small vesicles (population 1). If the small vesicle fraction was evaluated together with the components with a high UV signal (presumably proteins), the average *R*_g_ was found to be ~25 nm, which is in good agreement with the size of population 1 as determined by batch DLS (*R*_h_ ≈ 15–35 nm; note that *R*_g_ of smaller population 1 could not be determined by batch SLS and therefore *R*_g_ cannot be directly compared). In general, the average *R_g_* values of highly polydisperse systems determined by SLS in batch mode are larger than those determined by SLS coupled to one of the separation systems, such as in AF4-MALS, since in the former case, much stronger scattering from the larger particles obscures the scattering from the smaller particles, which is a known limitation of the batch-mode SLS.

**Figure 5:**
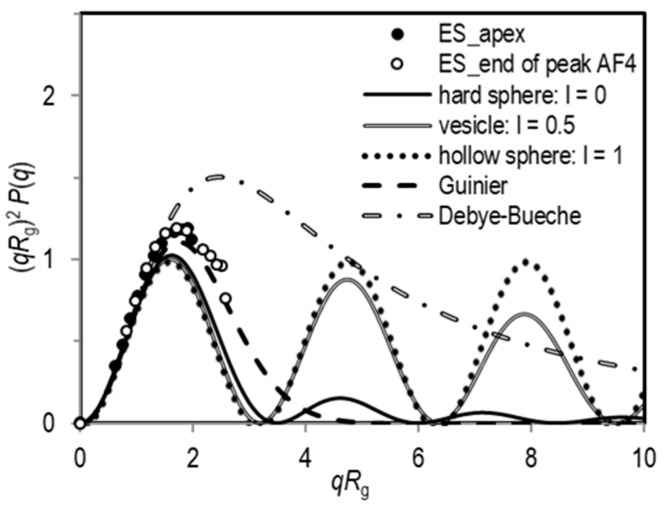
Kratky plots (*qR*_g_)^2^*P*(*q*) vs. *qR*_g_) were constructed for large particles eluted at a peak apex (32 min, full circles) and at ~35 min (open circles) in the AF4/UV-MALS fractogram of the filtered exosome standard (ES).

The SLS data acquired in the flow mode are represented also in the form of the Kratky plots (Figure 5). Kratky plots were constructed from the LS data of exosome fractions eluted at 32 and 35 min in the fractogram of exosome sample. In the available *qR*_g_ region, the data are in line with the form factor of spherical particles, indicating spherical shape of exosomes as well as their successful separation by size.

### 3.2. LS Size Characterization of Particles in Blood Plasma

The distribution of the particles in blood plasma and the EV isolates as obtained by batch DLS were most often tri-modal (representative distribution is shown in Figure 2). As previously described, the peak with *R*_h_ < 10 nm was assigned to the proteins (marked with 0), while the peaks 1 and 2 were assigned to the EVs. The *R*_g_ and *R*_h_ values pertaining to peak 1 (small vesicles—population 1) were 10–50 nm (diameter 20–100 nm) and those of peak 2 (large vesicles—population 2) were mostly between 100–150 nm (diameter ~200–300 nm). Sometimes, larger particles (*R*_h_ (*R*_g_) > 350 nm, not reported in the tables) were identified as well. They were attributed to aggregates or other large particles in trace amounts.

Similar scattering profiles observed in blood plasma samples, EV isolates, and exosome standards suggest that the complexity of plasma does not prevent the analysis by the approach used here (the separate analysis of two dynamic modes). Population 2 generally contributed a little more to the total LS intensity in EV isolates in comparison to the blood plasma (compare peak heights in *R*_h_ distributions reported in Figure 2). This was expected as isolates are supposed to be enriched in EVs. The number densities of particles in the EV isolates as detected by FCM were 1–7 × 10^6^ particles/mL (see representative FCM images in Figure S3 in SM). The lower limit of detection by FCM is the size of approximately 300 nm, meaning that only relatively large particles (belonging to population 2 or to even larger particles) were counted, whereas the method is blind for small particles (population 1), which were detected by both DLS and AF4. However, the FCM result shows that the vesicle suspensions were rather dilute. The small number densities resulting from FCM are in agreement with the mass- and number-weighted distributions, which can be calculated from the measured intensity-weighted DLS distributions by taking into account the spherical geometry of particles (see Equation S6 and Figure S1B in SM).

It is clearly seen from Figure 2, that if viscosity of water was used in Equation (2) for *R*_h_ calculation, the size distributions of population 2 in full blood plasma samples were systematically shifted to higher mean *R*_h_ values relative to the EV isolate prepared from the same blood plasma or to the exosome standard. The overestimation of *R*_h_ pertaining to population 2 in the full blood plasma was confirmed by simple dilution experiments (see results for the sample HD3 in Table S4, SM). The dilution of a thawed HD3 plasma sample leads to a decrease in *R*_h,0_ from 436 nm (undiluted sample) to 363 nm (1:1 dilution) and 286 nm (1:3 dilution). It is therefore suggested that the incorrect consideration of viscosity is the prime cause for this size shift. This assumption is investigated in more detail below.

### 3.3. Determination of the Medium Viscosity for Correct Characterization of the EV size by DLS in Full Blood Plasma Samples

In DLS, the diffusion coefficient *D* of particles is measured and therefrom *R*_h_ is calculated by using the Stokes-Einstein equation (Equation (2)). In this calculation, a spherical shape of particles is assumed. However, the calculated *R*_h_ values largely depend on the definition of the medium, where the particles diffuse, and along with this on the viscosity. The actual concentration of diffusing particles for which *D* is being measured is also important. It needs to be sufficiently low, so that inter-particle interactions can be ignored. For our samples, this was confirmed by FCM (see above). The viscosity issue, on the other hand, is often completely ignored in the determination of EVs’ size by DLS. Most often, water viscosity is taken into account in the *R*_h_ evaluation, which is a big simplification. In an aim to determine a more appropriate viscosity of the medium for the analysis of vesicles’ *R*_h_ in complex samples such as blood plasma, several ultracentrifugation (UC) experiments were performed. It was presumed that the measurements of dynamic viscosities of separate plasma fractions, along with the evaluation of their protein and particle contents, can give answers about the appropriate viscosity value to be used in the Stokes-Einstein equation in derivation of *R*_h_ of the EV-sized particles in blood plasma samples. The details of various ultracentrifugation procedures are reported in Section S2.2.1 and Section S2.2.2 in SM.

In DLS, the diffusion coefficient *D* of particles is measured and therefrom *R*_h_ is calculated by using the Stokes-Einstein equation (Equation (2)). In this calculation, a spherical shape of particles is assumed. However, the calculated *R*_h_ values largely depend on the definition of the medium, where the particles diffuse, and along with this on the viscosity. The actual concentration of diffusing particles for which *D* is being measured is also important. It needs to be sufficiently low, so that inter-particle interactions can be ignored. For our samples, this was confirmed by FCM (see above). The viscosity issue, on the other hand, is often completely ignored in the determination of EVs’ size by DLS. Most often, water viscosity is taken into account in the *R*_h_ evaluation, which is a big simplification. In an aim to determine a more appropriate viscosity of the medium for the analysis of vesicles’ *R*_h_ in complex samples such as blood plasma, several ultracentrifugation (UC) experiments were performed. It was presumed that the measurements of dynamic viscosities of separate plasma fractions, along with the evaluation of their protein and particle contents, can give answers about the appropriate viscosity value to be used in the Stokes-Einstein equation in derivation of *R*_h_ of the EV-sized particles in blood plasma samples. The details of various ultracentrifugation procedures are reported in Section S2.2.1 and Section S2.2.2 in SM.

**Figure 6:**
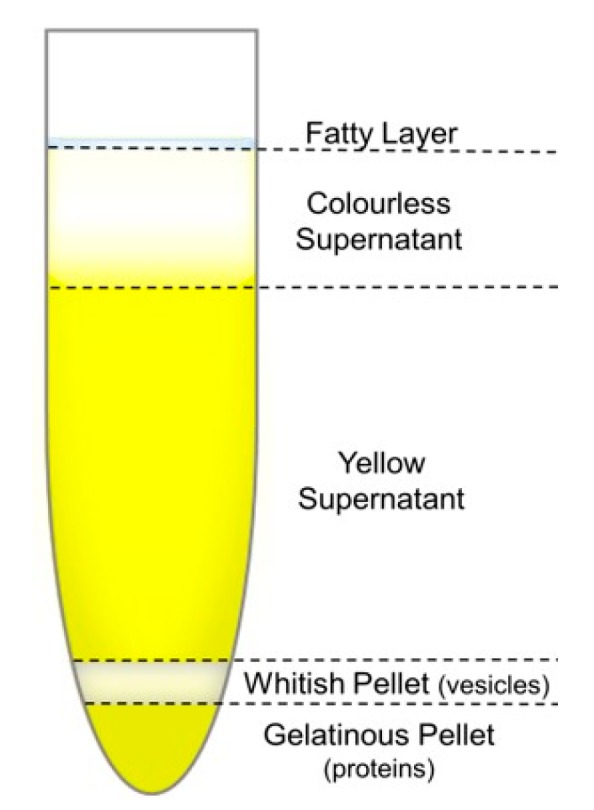
The fractions of the blood plasma sample after 8 h of ultracentrifugation at 100,000× *g*.

In the preliminary sedimentation test using 4 cycles (1 h each) of centrifugation at 100,000× *g* (results in Section S2.2.1.), the blood plasma sample remained almost unchanged. After 8 h at 100,000× *g*, the plasma separated into 5 fractions as shown in Figure 6 (final stage with some more details is represented in Figure S6). The elaboration of the diluted samples (dilutions 1:1 or 1:2 were tested; pictures not shown) resulted in the same final state, implying that the separation limit in regard to the applied conditions was reached. 

A brief evaluation of protein contents in the obtained fractions by SDS-PAGE and UV-vis spectrometry (see results in Figure S7 and Table S8, SM) showed that the majority of proteins precipitated into the gelatinous bottom pellet, the yellow supernatant fraction contained similar concentration of proteins as the initial blood plasma sample, while the colourless supernatant was largely protein-depleted. No apparent qualitative differences between the proteins in the fractions were noticed in SDS-PAGE separation, except for the colourless supernatant, which contained proteins that could be assigned to the low-density lipoproteins (LDL and VLDL).

The viscosities of the initial blood plasma sample (*η* = 1.47 mPa/s; c.f. Table 2), the obtained liquid fractions after UC separation, i.e., of the colourless (*η* = 0.94 mPa/s), the yellow supernatant (*η* = 1.32 mPa/s; c.f. Table 2) and of their mixture (*η* = 1.21 mPa/s), along with the viscosity of triple distilled water (*η*_0_ = 0.90 mPa/s) and of a 70 mg/mL HSA solution in PBS (*η* = 1.19 mPa/s) were measured. All these values are reported in Table S7. The viscosities of the pellets could not be measured by the capillary Ubbelohde viscometer.

According to the DLS results, the majority of the vesicles gathered in the upper (whitish) section of the pellet. The whitish and gelatinous pellets were analysed by DLS/SLS after re-suspending them in PBS. The resulting PBS suspensions were rather dilute; therefore, no viscosity correction was made in *R*_h_ evaluation of particles in this case (c.f. designations NC in the last column of Table 2).

Accounting for the dynamic viscosity of each fraction, the corrected *R*_h,90_ values, designated as *R*_h,90_^corr^ (see the penultimate column in Table 2), of the EV populations in different samples became very similar to each other, i.e., *R*_h,90_^corr^ were between 40 and 46 nm (compare this to the range between 40 and 75 nm for the uncorrected *R*_h,90_ values).

As the vesicle-free plasma could not be obtained by UC, an artificial substitute was prepared, i.e., a HSA (as the most abundant plasma protein) suspension in PBS at the usual concentration of proteins in blood plasma (70 mg/mL). It was assumed that it could represent a relevant imitate of the medium in which vesicles in blood plasma diffuse. Interestingly, the dynamic viscosity of the total supernatant (*η* = 1.21 mPa/s; c.f. Table S7), which is a mixture of the colourless and yellow supernatants, was very similar to that of the HSA suspension. The viscosity value of 1.2 mPa/s was thus taken as an approximation for *η* while *R*_h_ of EVs in blood plasma was calculated by using Equation (2).

### 3.4. Shape Characterization of Particles by DLS/SLS

In cases when *R*_g_ was successfully determined (mainly for the population 2 particles, while sizes of the population 1 particles were usually too small and *R*_g_ could not be obtained; see above), the shape parameter *ρ* (= *R*_h,0_/*R*_g_) was calculated to get the structural information about the analysed particles. The values of approximately 1 (average *ρ* = 1.02 with a standard deviation of ± 0.04; c.f. Table S1, SM) were determined for the larger particles (population 2) in exosome standards by considering *η*_0_ = 0.90 mPa/s in the calculation of *R*_h_, as they were analysed in very dilute aqueous suspensions. Taking into account the uncertainty of DLS and SLS (around 5% for both) and the polydispersity of particles, this *ρ* value agrees very well with the theoretical one for monodisperse spherical shells (*ρ* = 1.0).

In blood plasma and the EV isolates derived from it, the average *ρ* of the populations with similar average size (calculated by proposing the viscosity of the medium in blood plasma samples to be *η* = 1.2 mPa/s at 25 °C; see above, and *η*_0_ = 0.90 mPa/s in case of EV isolates, which were very dilute suspensions) was approaching 1 as well, but with some wider variance (0.69 < *ρ* < 1.16; c.f. Tables S2–S4, SM). This spread of the *ρ*-values was expected as the populations were more heterogeneous in those samples and, importantly, the viscosity of blood plasma of different persons is expected to be different, which was ignored in our analysis.

## 4. Discussion

The combination of DLS and SLS techniques have been used to analyse samples containing EVs. The size distributions and relative ratios between populations of particles in the samples of exosome standard, blood plasma and EV isolates from blood plasma of healthy donors were determined. The additional information on the size and shape of EVs was obtained from the angular dependency of the measured LS parameters by considering separately the dynamic modes, the effect of polydispersity on the data analysis, and by using the appropriate viscosity coefficient of the medium (background fluid) in which EVs were characterized.

### 4.1. Size Characterization

The *R*_h_ value crucially depends on the assumption of the medium viscosity. Despite this very obvious fact, viscosity is still poorly understood and thus often an ignored parameter in the LS analysis of biological and other complex samples in general. It is generally accepted that the determined *R*_h_ is correct if the concentration of the analysed particles (EVs) is sufficiently low so that inter-particle interactions can be ignored. The eventual interactions between the particles would contribute also to the viscosity they experience. If the concentration of EVs is low, as is the case for our samples, the relevant viscosity is that of the background fluid. The questions are: What is the background fluid in case of blood plasma samples? Which components of the blood plasma constitute the medium relevant for the analysis of a specific population of particles in them? Plasma namely contains water, many small organic compounds like amino acids, vitamins, minerals, and also larger solutes, macromolecules such as proteins, protein complexes, lipoproteins, lipid drops, EVs etc. An appropriate criterion is needed to distinguish each component of a suspension as being a particle or a constituent of the medium.

Unfortunately, the question of the size-viscosity relationship in the DLS measurements cannot be resolved by simply adding an internal size-standard into the complex sample, such as blood plasma. There are various factors that may compromise such result: e.g., the hydration layer and possible protein shell formation, stacking of particles (realistic increase of the standard particle size), the inter-particle forces (contributing to or competing with the Brownian motion of particles), the aggregation of particles (introducing the large-particle interference to the measurement), and finally also the specific, particle size-dependent [48] (effective) viscosity of the medium.

Taking an example of the fractioned blood plasma sample from this study (Table 2), it can be seen that by using the measured viscosity of a particular fraction, the resulting *R*_h_ values of larger particles become very similar in all fractions. This result suggests a large impact of viscosity on the calculated *R*_h_ and implies that similar (possibly the same) particles are perceived in different fractions. However, it should be emphasized that the viscosity measured for the whole sample is not directly applicable to obtain accurate *R*_h_ values of all particles present in it, as discussed in the literature as well [49]. As can be observed from the shift in size distributions of the samples in Figure 2, the correction that seems to be appropriate for the vesicles (peak 2, and conditionally peak 1) is not equally suitable to determine the correct size of the smaller particles (peak 0). A plasma suspension may be compared to the crowded systems studied in relation to DNA condensation induced by polymers. Holyst et al. [48] have argued that effective viscosity (the viscosity experienced by a particular particle) in a crowded system depends on the size of that particle. When the molecule is significantly smaller in comparison to other components, it experiences viscosity equivalent to dilute aqueous solutions. On the other hand, when the molecule is comparable in size with other components, the length scale of mutual interaction between components dictates the viscosity it encounters. Consequently, the viscosity appropriate for larger particles may not be directly applicable for the size determination of smaller particles despite being analysed in the same suspension. And therefore, the *R*_h_ values (or size parameters in general) that are derived by one common arbitrary viscosity value must be interpreted carefully.

It has been assumed that the relevant medium for EV analysis in blood plasma would be vesicle-free plasma, a solution of all other components except EVs. This could not be achieved by UC in this study due to the slow sedimentation rate of vesicles and extensive precipitation of proteins (into the whitish and gelatinous pellets stuck at the bottom of the UC tube; c.f. Figure 6) during prolonged ultracentrifugation times. Therefore, an artificial substitute was prepared, a 70 mg/mL HSA solution in the PBS buffer. Interestingly, the viscosity of the HSA solution happened to be very similar to that of the viscosity of the total supernatant after plasma separation (whitish and colourless liquid fractions; c.f. Figure 6). The differences in size distributions between plasma samples and the corresponding EV isolates were diminished after such correction. It is therefore supposed that it represents the most likely vesicle-free background fluid for EVs characterization in blood plasma. Further support to this choice is the fact that the concentration of EVs in blood plasma is usually low and their size is relatively large in comparison with other components. However, the blood plasma composition depends significantly on many (individual and external) factors and to obtain the most accurate results, each sample should be considered individually.

In complex samples, the model based on the simple Stokes-Einstein equation may not meet all the required conditions for its relevance, and does not suffice for a very precise investigation of all individual populations. The model should be improved by taking into account the properties of the EV systems.

### 4.2. Identification of Subpopulations of Extracellular Vesicles in Blood Plasma

In all the samples studied here, the population of larger particles (population 2) contributed the major part to the total scattered light intensity (for example 70–95% in the exosome standards; Figure S1). Considering the fact that population 1 represents the number and mass majority of the EV-sized particles (see Figure S1B) in the exosome standard suspensions used in this study, it is suggested that information about exosomes is carried by the population of small particles (population 1, mean *R*_h_ ≈ 10–35 nm). The population 2 (larger particles) found in the samples from this study (both exosome standard and blood plasma) may be assigned to microvesicles and/or possibly exosome aggregates.

As free proteins are obviously present in these samples according to the AF4 results, but not always separately detected by the batch DLS/SLS, it is supposed that the *R*_h_ values of exosomes in protein rich samples analysed by the batch DLS/SLS might be underestimated due to the poor resolution of the method for the analysis of small particles. Some overlap of protein fractions with exosomes was also reported previously by Sitar et al. [39], who analysed an exosome standard isolated from the supernatant of the lymphoblastoid B cell line culture. By TEM, the small vesicles (with approximate diameter 15 nm) along with many protein aggregates were observed and by AF4/UV-MALS, one broad peak with low UV absorbance and a mean particle radius over 100 nm was determined. The latter value is similar to the size of larger particles in this study.

On the other hand, the *R*_h_ and *R*_g_ values of the large particles are probably overestimated by the batch DLS because the intensity in polydisperse populations is weighted towards large particles. In agreement with this, the size parameters obtained for the exosome standard by AF4/UV-MALS are generally smaller than those obtained by the batch DLS/SLS.

### 4.3. Structural Characterization

One of the very basic disadvantages of DLS is that it cannot distinguish between the case of multimodality originating from completely different populations, and a polydisperse population of particles and their aggregates [50]. It was previously observed in various studies that isolated vesicles are likely to undergo oligomerisation and aggregation [2,51]. Various handling procedures, like filtering of the sample or trehalose addition, did not affect the peak for larger particles (see AF4/UV-MALS results in Figure 4 and Figure 5 and Table 1 and DLS/SLS results for population 2 in Tables S1–S4) to an extent that would permit the distinction between exosome aggregates or large vesicles.

Some aggregates were observed in the AFM micrographs (see Figure 1). This can also be a result of sample preparation for microscopy. The AFM was employed to reveal potential morphological changes of vesicles in exosome standards caused by filtration (Figure 1A,B). The similar spheroidal shape of particles was observed in both AFM micrographs so as the grouping of the vesicles into aggregates. The particles’ size in filtered and unfiltered ES was similar, but particles in the filtered sample seemed slightly deformed. It has to be noted, however, that the area inspected by AFM was very small and may not be representative for the whole sample. Besides, the reproducible preparation of samples for AFM is difficult. The above differences may be associated with these particular experimental procedures in AFM. Finally, this study concluded that AFM results confirmed our anticipations obtained from scattering techniques, i.e., the presence of small and large particles, large polydispersity of populations and possible aggregation of vesicles.

A more detailed structural characterization of EVs has been performed by using the Kratky plot presentation. As far as the authors could ascertain, this approach was used for the first time in this study to describe the shape of EVs in blood plasma. In theory, the Kratky plot can display the differences in the internal structure of monodisperse spherical particles [47], as their form factor *P*(*q*) could exhibit characteristic oscillations (see calculated curves in Figure 3 and Figure 5, Figure S2). However, the aggregation and polydispersity have a considerable influence on the measured *P*(*q*). The deviations of the experimental data from the theoretical spherical-shape form factors and the loss of these oscillations with increasing angles are expected [45,46,47]. The experimental data in the Kratky plot curve may exhibit positive deviations at higher *qR*_g_, which is often observed for the herein studied samples (see for example the data in Figure 3). Besides, the presence of aggregates of EVs with inhomogeneous mass distribution could explain the suitability of the Debye-Bueche (or sometimes even Guinier) function for the description of the experimental data of our samples.

The shape parameters *ρ* of the population 2 particles in the plasma and EV isolates were dispersed around the value expected for spherical shapes, i.e., between the value for a hard sphere (*ρ* = 0.78), hollow sphere (*ρ* = 1), and slightly elongated oval structures (*ρ* = 1.2). In some cases, the lower values (*ρ* < 0.8) typical for microgel-like particles with a core-shell structure were obtained as well. This is in agreement with previous results [12] that the EV isolates are heterogeneous with respect to shape. The EVs that lack the internal structure attain the shape according to the minimum of the free energy of the membrane at a given area and volume. The lateral distribution of membrane constituents, their intrinsic shapes and interactions between them also affect the local and the global membrane curvature. Furthermore, the proteins and lipids in the membrane can be glycosylated and the attached sugar chains can greatly increase the hydrodynamic radius, while they have a smaller effect on the radius of gyration, leading to smaller *ρ* ratio. For example, low *ρ* values (*ρ* ≈ 0.6) were previously determined for mucine-rich vesicles of respiratory epithelium [24].

### 4.4. Further Considerations

DLS may give averaged results when analysing polydisperse and multimodal samples. Some peaks in the calculated distributions may be lost and/or shifted. The programs for evaluating *R*_h_ from the DLS measurements usually yield one of the (many) possible solutions of the mathematical transformation of the correlation function. CONTIN, which is generally accepted as a convenient approach for the treatment of correlation functions from multimodal and polydisperse systems, does not distinguish between the populations that differ in size by less than a factor of 2 [32]. It was previously reported that in the analysis of light scattering from samples with monomodal but very wide size distribution (i.e., very polydisperse samples), inverse transformation procedures (such as the inverse Laplace transformation employed in the CONTIN approach) tend to yield bimodal distributions [25]. This was also shown in the study of Varenne et al. [52] in which the polydisperse population of poly(isobutyl cyanoacrylate) nanoparticles (60–600 nm, as determined by AFM) was analysed by LS. Two to three populations were recognized by DLS and SLS in that study, and *R*_g_ (= 63.8 nm) of the smaller one was approximately twice as large as its *R*_h_ (= 35 nm, resulting in *ρ* ≈ 1.8), while *R*_g_ and *R*_h_ of the larger population were very similar to each other (*R*_g_ = 199 nm and *R*_h_ = 195 nm, leading to *ρ* ≈ 1). Those results are comparable to the ones obtained in this study. Namely, two populations of EVs were within the size range 10–500 nm. The population of smaller EVs yielded *ρ* = 1.3–1.8 and the population of larger ones yielded *ρ* ~ 1 (see Tables S2–S4, SM). Note that there was no viscosity correction of *R*_h_ in case of smaller particles. Altogether, it seems possible that such result may arise mainly as a consequence of sample’s heterogeneity.

In the studied samples, a local minimum of the scattered light intensity at angles around 110° (*qR*_g_ ≈ 3) was often observed. This can be an attribute of spherical structures (the characteristic oscillations with a minimum at *qR*_g_ = 33.5). On the other hand, it may also indicate a presence of larger particles [53] having size comparable to the wavelength of the He-Ne laser (*λ*_0_ = 633 nm), and therefore manifest a broad size distribution of particles. The high polydispersity of the population 2 is expressed through the deviations of *P*(*q*) function from the form factor of a vesicle (see Figure 3). A population of particles with similar *D* and therefore *R*_h_, can include particles of various shapes and origins (e.g., vesicles, small vesicle aggregates, lipoproteins, large protein aggregates, etc.). As mentioned above, the heterogeneity of the sample may have a profound effect on the results presented. A possibility should be considered that populations of EVs are not small cells which may retain their identity and properties during the processing of samples. Instead, the fragmentation and fusion of the particles with the redistribution of membranous constituents is likely to take place in the samples [12]. The interpretation of the LS measurements could be largely improved by taking into account particularities of EV samples to yield a plausible and relevant method for assessment of the EV samples.

## 5. Conclusions

Using the He-Ne laser (*λ*_0_ = 632.8 nm), the populations of vesicles were analysed by static and dynamic light scattering. The sub-population of small vesicles (population 1, *R*_h_ < 35 nm) could not be well characterized due to their small size and the possible overlap of their peak in *R*_h_ distribution with the population of proteins. A light source of shorter wavelength might overcome this difficulty. On the other hand, the sub-population of larger vesicles (population 2, *R*_h_ ≈ 100–150 nm) could be reliably characterized. Generally, for accurate size (*R*_h_) and subsequent structural characterization of EVs, it is crucial to use the appropriate viscosity of the medium. In this study, dynamic viscosities of the different blood plasma fractions (obtained by ultracentrifugation) were determined and finally, a solution of proteins and electrolytes was identified to be a rough but suitable approximation of a background fluid for EVs’ *R*_h_ estimation in blood plasma. The value of *η* = 1.2 mPa/s (at 25 °C) was found suitable for the calculation of parameters in samples of blood plasma, whereas in the EV isolates as well as in the exosome standard, the viscosity of water (*η* = 0.9 mPa/s at 25 °C) was appropriate. The obtained *ρ* of larger particles was 0.94–1.1 in the exosome standards and 0.7–1.2 in the blood plasma samples of healthy donors. Considering the variability of biological material and complexity of the samples (in particular polydispersity), these results are in a fairly good agreement with the theoretical *ρ* value (*ρ* ~ 1) for hollow spherical shapes. Through complementation of several experimental methods, this study showed that by DLS and SLS techniques, some valuable information can be extracted from the analysis of EVs in blood plasma, not only in the EV isolates. The advantage of DLS and SLS is also that the sample can be re-used. However, a detailed investigation of the laws of effective viscosity experienced by various particles in the complex suspensions would be beneficial in the LS field in general and certainly in the analysis of extracellular vesicles. This technique has a good potential in diagnostics, and when optimized, it may be advantageous for quality assessment and monitoring of purification, sample/treatment preparation, and the control of sample changes upon storage.

## Figures and Tables

**Table 1:** Radius of gyration (*R*_g_) as determined by AF4/UV-MALS as well as *R*_h,90_ and *R*_g_ derived from batch DLS/SLS for different populations in the exosome standard.

		*AF4-MALS*	*Batch DLS/SLS*	
Sample		*R*_g_ (nm)	*R_h,90_^#^* (nm)	*R_g_* (nm)
unfiltered ES	larger EVs (*population 2*)	93	119	152
	smaller EVs (*population 1*)	~40	20	ND *
	Average of short-time eluted particles; i.e., components with high UV signal and smaller EVs (*population 1*)	~25		
filtered ES	larger EVs (*population 2*)	84	101	138
	smaller EVs (*population 1*)	~40	19	ND *
	Average of short-time eluted particles; i.e., components with high UV signal and smaller EVs (*population 1*)	~25		

^#^ These *R*_h,90_ values were calculated from Equation (2) by taking into account the viscosity of water (*η*_0_ = 0.90 mPa/s). * ND = not defined.

**Table 2:** The results of the dynamic light scattering (DLS) analysis of plasma fractions formed during ultracentrifugation: the hydrodynamic radius at 90° (*R*_h,90_) and the contribution of individual peaks to the total LS intensity (I in %). The values for populations that presumably apply to extracellular vesicles are written in bold. In the penultimate column, the *R*_h,90_ values are corrected by taking into account the measured viscosity (given in the last column) of the fraction in question and are designated as *R*_h,90_^corr^.

	Peak	*I* [%]	*R*_h,90_ [nm]	*R*_h,90_^corr^ [nm]	Measured Viscosity, *η* [mPa/s]
Initial plasma sample *I* (total) = 792 kHz/mW	0	5 ^(a)^	3 ^(a)^	3 ^(a)^	1.47
	1	31	19	12	
	2	**64**	**75**	**46**	
Colourless supernatant *I* (total) = 904 kHz/mW	1	46	21	20	0.94
	2	54 ^(b)^	55 ^(b)^	52 ^(b)^	
Yellow supernatant *I* (total) = 746 kHz/mW	0	3 ^(a)^	2 ^(a)^	2 ^(a)^	1.32
	1	31	14	9	
	2	**64**	**62**	**42**	
Whitish pellet re-suspended in PBS *I* (total) = 490 kHz/mW	1	17 ^(a)^	7 ^(a)^	7 ^(a)^	NC ^c)^
	2	**77**	**44**	**44**	
Gelatinous pellet re-suspended in PBS *I* (total) = 113 kHz/mW	0	50 ^(a)^	7 ^(a)^	7 ^(a)^	NC ^(c)^
	1	**49**	**40**	**40**	
			

^(a)^ The peak was assigned to proteins and the *R*_h,90_ value was not corrected. ^(b)^ The peak was assigned to lipoproteins/lipid drops; the *R*_h,90_ value was corrected. ^(c)^ No correction (NC) was done for the samples of the pellets (the bottom two fractions; c.f. Figure 6) that were re-suspended in PBS. The viscosity coefficient of water (*η*_0_ = 0.90 mPa/s) was used in *R*_h_ evaluation.

## Supplementary Materials

The following are available online at https://www.mdpi.com/2073-4409/8/9/1046/s1, Scheme S1: Vesicle structure parameters for the form factor derivation, Figure S1: Dynamic light scattering data transformation–from the measured correlation function to the size distribution, Figure S2: Kratky plot representation of the static light scattering data–some theoretical functions, Figure S3: Gating strategy for the analysis of EVs by FCM, Figure S4: Measured correlation function (G2(t)), calculated g1(t) and intensity distributions for an exosome standard, blood plasma and EV isolate from it, Figure S5: Comparison of the angular dependency of the hydrodynamic radii, Rh, and the reciprocal scattered light intensity for the population 2 in the unfiltered and filtered exosome standard, Figure S6: Photography of blood plasma after 8-h of ultracentrifugation at 100 000× g, Figure S7: SDS-PAGE of blood plasma and its fractions obtained after 8-h of ultracentrifugation at 100 000× g, Table S1: Results of the DLS and SLS analysis of EVs in the exosome standard, Tables S2–S4: Results of the DLS and SLS analysis of EVs in blood plasma of three healthy donors, Table S5: Density and dynamic viscosity of the fractions obtained in the preliminary blood plasma sedimentation test, Table S6: Results of DLS analysis for fractions obtained in the preliminary blood plasma sedimentation test, Table S7: Dynamic viscosity of blood plasma and its fractions after 8-h of ultracentrifugation at 100 000× g, Table S8: Protein contents of plasma fractions obtained after 8-h ultracentrifugation at 100 000× g.

## References

[1] Simpson R.J., Mathivanan S. (2012). Extracellular Microvesicles: The Need for Internationally Recognised Nomenclature and Stringent Purification Criteria. J. Proteomics Bioinform..

[2] Linares R., Tan S., Gounou C., Arraud N., Brisson A.R. (2015). High-speed centrifugation induces aggregation of extracellular vesicles. J. Extracell. Vesicles.

[3] Johnsen K.B., Gudbergsson J.M., Andresen T.L., Simonsen J.B. (2019). What is the blood concentration of extracellular vesicles? Implications for the use of extracellular vesicles as blood-borne biomarkers of cancer. Biochim. Biophys. Acta.

[4] Černe K., Kobal B., Aleš I. (2012). Chapter Eight-Implications of Microvesicle and Cell Surface Protein Shedding for Biomarker Studies, Cancerogenesis, and Therapeutic Target Discovery in Ovarian Cancer. Advances in Planar Lipid Bilayers and Liposomes.

[5] Yáñez-Mó M., Siljander P.R.M., Andreu Z., Zavec A.B., Borràs F.E., Buzas E.I., Buzas K., Casal E., Cappello F., Carvalho J. (2015). Biological properties of extracellular vesicles and their physiological functions. J. Extracell. Vesicles.

[6] Muller L., Hong C.S., Stolz D.B., Watkins S.C., Whiteside T.L. (2014). Isolation of biologically-active exosomes from human plasma. J. Immunol. Methods.

[7] Witwer K.W., Buzas E.I., Bemis L.T., Bora A., Lasser C., Lotvall J., Nolte-’t Hoen E.N., Piper M.G., Sivaraman S., Skog J. (2013). Standardization of sample collection, isolation and analysis methods in extracellular vesicle research. J. Extracell. Vesicles.

[8] van der Pol E., Boing A.N., Harrison P., Sturk A., Nieuwland R. (2012). Classification, functions, and clinical relevance of extracellular vesicles. Pharmacological reviews.

[9] Zeringer E., Barta T., Li M., Vlassov A.V. (2015). Strategies for Isolation of Exosomes. Cold Spring Harb. Protoc..

[10] Lacroix R., Judicone C., Mooberry M., Boucekine M., Key N.S., Dignat-George F. (2016). Standardization of pre-analytical variables in plasma microparticle determination: Results of the International Society on Thrombosis and Haemostasis SSC Collaborative workshop. J. Thromb. Haemost..

[11] Štukelj R., Schara K., Bedina-Zavec A., Šuštar V., Pajnič M., Pađen L., Krek J.L., Kralj-Iglič V., Mrvar-Brečko A., Janša R. (2017). Effect of shear stress in the flow through the sampling needle on concentration of nanovesicles isolated from blood. Eur. J. Pharm. Sci..

[12] Šuštar V., Bedina-Zavec A., Štukelj R., Frank M., Bobojević G., Janša R., Ogorevc E., Kruljc P., Mam K., Šimunič B. (2011). Nanoparticles isolated from blood: A reflection of vesiculability of blood cells during the isolation process. Int. J. Nanomed..

[13] Corrado C., Raimondo S., Chiesi A., Ciccia F., De Leo G., Alessandro R. (2013). Exosomes as intercellular signaling organelles involved in health and disease: Basic science and clinical applications. Int. J. Mol. Sci..

[14] Ko J., Carpenter E., Issadore D. (2016). Detection and isolation of circulating exosomes and microvesicles for cancer monitoring and diagnostics using micro-/nano-based devices. Analyst.

[15] Kätzel U., Vorbau M., Stintz M., Gottschalk-Gaudig T., Barthel H. (2008). Dynamic Light Scattering for the Characterization of Polydisperse Fractal Systems: II. Relation between Structure and DLS Results. Part. Part. Syst. Char..

[16] Shibayama M., Karino T., Okabe S. (2006). Distribution analyses of multi-modal dynamic light scattering data. Polymer.

[17] Schärtl W. (2007). Light Scattering from Polymer Solutions and Nanoparticle Dispersions.

[18] Kratochvil P., Huglin M.B. (1972). Particle scattering functions. Light scattering from polymer solutions.

[19] Lawrie A.S., Albanyan A., Cardigan R.A., Mackie I.J., Harrison P. (2009). Microparticle sizing by dynamic light scattering in fresh-frozen plasma. Vox Sang..

[20] Erdbrugger U., Lannigan J. (2016). Analytical challenges of extracellular vesicle detection: A comparison of different techniques. Cytometry A.

[21] Pencer J., Hallett F.R. (2003). Effects of Vesicle Size and Shape on Static and Dynamic Light Scattering Measurements. Langmuir.

[22] Petersen K.E., Manangon E., Hood J.L., Wickline S.A., Fernandez D.P., Johnson W.P., Gale B.K. (2014). A review of exosome separation techniques and characterization of B16-F10 mouse melanoma exosomes with AF4-UV-MALS-DLS-TEM. Anal. Bioanal. Chem..

[23] Palmieri V., Lucchetti D., Gatto I., Maiorana A., Marcantoni M., Maulucci G., Papi M., Pola R., De Spirito M., Sgambato A. (2014). Dynamic light scattering for the characterization and counting of extracellular vesicles: A powerful noninvasive tool. J. Nanopart. Res..

[24] Kesimer M., Gupta R. (2015). Physical characterization and profiling of airway epithelial derived exosomes using light scattering. Methods.

[25] Fischer K., Schmidt M. (2016). Pitfalls and novel applications of particle sizing by dynamic light scattering. Biomaterials.

[26] Han C., Sun X., Liu L., Jiang H., Shen Y., Xu X., Li J., Zhang G., Huang J., Lin Z. (2016). Exosomes and Their Therapeutic Potentials of Stem Cells. Stem Cells Int..

[27] Bruning B., Stehle R., Falus P., Farago B. (2013). Influence of charge density on bilayer bending rigidity in lipid vesicles: A combined dynamic light scattering and neutron spin-echo study. Eur Phys. J. E. Soft Matter..

[28] Zhang W., Peng P., Kuang Y., Yang J., Cao D., You Y., Shen K. (2016). Characterization of exosomes derived from ovarian cancer cells and normal ovarian epithelial cells by nanoparticle tracking analysis. Tumour Biol..

[29] Bhattacharjee S. (2016). DLS and zeta potential-What they are and what they are not?. J. Controlled Release.

[30] Filipe V., Hawe A., Jiskoot W. (2010). Critical evaluation of Nanoparticle Tracking Analysis (NTA) by NanoSight for the measurement of nanoparticles and protein aggregates. Pharm Res..

[31] Sitar S., Aseyev V., Kogej K. (2014). Differences in association behavior of isotactic and atactic poly(methacrylic acid). Polymer.

[32] Sitar S., Aseyev V., Kogej K. (2014). Microgel-like aggregates of isotactic and atactic poly(methacrylic acid) chains in aqueous alkali chloride solutions as evidenced by light scattering. Soft Matter.

[33] Brown W., Brown W. (1993). Dynamic light scattering: The method and some applications.

[34] Bosch S., de Beaurepaire L., Allard M., Mosser M., Heichette C., Chretien D., Jegou D., Bach J.M. (2016). Trehalose prevents aggregation of exosomes and cryodamage. Sci Rep..

[35] Klucker R., Munch J.P., Schosseler F. (1997). Combined Static and Dynamic Light Scattering Study of Associating Random Block Copolymers in Solution. Macromolecules.

[36] Raspaud E., Lairez D., Adam M., Carton J.P. (1994). Triblock Copolymers in a Selective Solvent. 1. Aggregation Process in Dilute Solution. Macromolecules.

[37] Tarassova E., Aseyev V., Filippov A., Tenhu H. (2007). Structure of poly(vinyl pyrrolidone)–C70 complexes in aqueous solutions. Polymer.

[38] Kogej K., Šorl S. (2017). Temperature dependence of solution properties of anionic polyelectrolyte-cationic surfactant complexes in ethanol. J. Mol. Liq..

[39] Sitar S., Kejzar A., Pahovnik D., Kogej K., Tusek-Znidaric M., Lenassi M., Zagar E. (2015). Size characterization and quantification of exosomes by asymmetrical-flow field-flow fractionation. Anal. Chem..

[40] Zhang H., Lyden D. (2019). Asymmetric-flow field-flow fractionation technology for exomere and small extracellular vesicle separation and characterization. Nat. Protoc..

[41] Gumz H., Boye S., Iyisan B., Krönert V., Formanek P., Voit B., Lederer A., Appelhans D. (2019). Toward Functional Synthetic Cells: In-Depth Study of Nanoparticle and Enzyme Diffusion through a Cross-Linked Polymersome Membrane. Adv. Sci..

[42] Elvang P.A., Stein P.C., Bauer-Brandl A., Brandl M. (2017). Characterization of co-existing colloidal structures in fasted state simulated fluids FaSSIF: A comparative study using AF4/MALLS, DLS and DOSY. J. Pharm. Biomed. Anal..

[43] Debye P., Bueche A.M. (1949). Scattering by an Inhomogeneous Solid. J. Appl. Phys..

[44] Debye P., Bueche F. (1952). Distribution of Segments in a Coiling Polymer Molecule. J. Chem. Phys..

[45] Savin G., Burchard W. (2004). Uncommon Solution Behavior of Poly(N-vinylimidazole). Angular Dependence of Scattered Light from Aggregates in Ethanol. Macromolecules.

[46] Stauch O., Schubert R., Savin G., Burchard W. (2002). Structure of Artificial Cytoskeleton Containing Liposomes in Aqueous Solution Studied by Static and Dynamic Light Scattering. Biomacromolecules.

[47] Ruckdeschel P., Dulle M., Honold T., Förster S., Karg M., Retsch M. (2016). Monodisperse hollow silica spheres: An in-depth scattering analysis. Nano Res..

[48] Holyst R., Bielejewska A., Szymanski J., Wilk A., Patkowski A., Gapinski J., Zywocinski A., Kalwarczyk T., Kalwarczyk E., Tabaka M. (2009). Scaling form of viscosity at all length-scales in poly(ethylene glycol) solutions studied by fluorescence correlation spectroscopy and capillary electrophoresis. Phys. Chem. Chem. Phys..

[49] Plantz E. Brownian Motion, Dynamic Light Scattering and the Use of Viscosity Or…Would You Argue with Albert Einstein about how to use Viscosity in the DLS Formula?. https://www.microtrac.com/MTWP/wp-content/uploads/2012/10/Microtrac-Application-Notes-Arguing-with-Einstein-How-to-Use-Viscosity-in-the-DLS-Formula.pdf.

[50] Fairhurst D., Weiner B. A Guide to Determination of Particle Size–Making an Effective and Reliable Measurement. http://www.brookhaveninstruments.com/pdf/theory/Determination%20of%20Particle%20Size.pdf.

[51] Thery C., Amigorena S., Raposo G., Clayton A. (2006). Isolation and characterization of exosomes from cell culture supernatants and biological fluids. Curr. Protoc. Cell Biol..

[52] Varenne F., Makky A., Gaucher-Delmas M., Violleau F., Vauthier C. (2016). Multimodal Dispersion of Nanoparticles: A Comprehensive Evaluation of Size Distribution with 9 Size Measurement Methods. Pharm. Res..

[53] Gao S., Shen J., Thomas J.C., Yin Z., Wang X., Wang Y., Liu W., Sun X. (2015). Effect of scattering angle error on particle size determination by multiangle dynamic light scattering. Appl. Optics.

