# Supplementary material for: Viscosity of Plasma as a Key Factor in Assessment of Extracellular Vesicles by Light Scattering
Source: Cells. 2019 Sep 6;8(9):1046. doi: 10.3390/cells8091046 (PMC6769602; doi:10.3390/cells8091046)
Supplement: Supplementary file 1 [file cells-08-01046-s001.pdf]

Supplementary Material for the Article

Viscosity of plasma as a key factor in assessment of extracellular vesicles by light scattering

Darja Božič[1,#], Simona Sitar[2], Ita Junkar[3], Roman Štukelj[4], Manca Pajnič[4], Ema Žagar[2], Veronika Kralj-Iglič[4], Ksenija Kogej*[1]

[1] Department of Chemistry and Biochemistry, University of Ljubljana, Faculty of Chemistry and Chemical Technology, Večna pot 113, SI-1000 Ljubljana, Slovenia; ksenija.kogej@fkkt.uni-lj.si

[2] Department of Polymer Chemistry and Technology, National Institute of Chemistry, Hajdrihova 19, SI-1001 Ljubljana, Slovenia; simona.sitar@ki.si, ema.zagar@ki.si

[3] Department of Surface Engineering and Optoelectronics, Josef Stefan Institute, Teslova 30, SI-1000 Ljubljana, Slovenia; ita.junkar@ijs.si

[4] Biomedical Research Group, University of Ljubljana, Faculty of Health Sciences, Zdravstvena pot 5, SI-1000 Ljubljana, Slovenia; roman.stukelj@zf.uni-lj.si, manca.pajnic@zf.uni-lj.si, veronika.kralj-iglic@fe.uni-lj.si

[#] Present address:
Biomedical Research Group, University of Ljubljana, Faculty of Health Sciences, Zdravstvena pot 5, SI-1000 Ljubljana, Slovenia and
Laboratory of Physics, University of Ljubljana, Faculty of Electrical Engineering, Tržaška cesta 25, SI-1000 Ljubljana, Slovenia; darja.bozic@fe.uni-lj.si

* Correspondence: ksenija.kogej@fkkt.uni-lj.si
Department of Chemistry and Biochemistry
Faculty of Chemistry and Chemical Technology
University of Ljubljana
Večna pot 113
SI-1000 Ljubljana
SLOVENIA
ksenija.kogej@fkkt.uni-lj.si
Tel.: +386-1-479-8538

S1 Methods

S1.1. Light Scattering.

S1.1.1 Dynamic light scattering.

Dynamic light scattering, DLS, enables the determination of the relaxation time, τ, relaxation rate, Γ, or diffusion coefficient, D, of particles by calculating the correlation function of the scattered electric field, $g_1(t)$, from the measured correlation function of the scattered light intensity, $G_2(t)$, and applying the relation

$$|g_1(t)| = e^{-t/\tau} = e^{-\Gamma t} = e^{-Dq^2 t} \qquad \text{(S1)}$$

In equation S1, q is the scattering vector, given as $q = (4\pi\, n_0/\lambda_0)\sin(\theta/2)$, where n_0 is the refractive index of the medium, λ_0 is the wave-length of light and θ is the angle of observation. The values of τ (or D) can be determined by the inverse Laplace transform program CONTIN developed by Provencher [1]. In case that samples are polydisperse, a multi exponential fit is used to fit the $g_1(t)$ function and determine the exponents. The result of such procedure is the distribution of intensity over τ (or Γ or D) for the species in solution, which is then converted into the intensity weighted size distribution (i.e. over the hydrodynamic radii, R_h) by means of the Stokes-Einstein equation

$$R_h = \frac{kT}{6\pi\eta D} \qquad \text{(S2)}$$

In equation S2, k is the Boltzmann constant, T the absolute temperature, and η the viscosity of the solvent.

For the herein studied samples, all size distributions were multimodal, exhibiting 2–4 peaks. The mean R_h values for the peaks determined at $\theta = 90°$ and their origin are reported in the main paper. An example of such multimodal distribution is shown in Figure S1, where each scattering species of the radius $R_{h,i}$ is represented by its relative contribution or amplitude, $A_i(\tau_i)$, to the total intensity of scattered light.

R_h values of particles that are larger than $\lambda_0/20$ or are polydisperse can significantly depend on the angle of observation. In some cases, peaks of different populations can merge into one(broad)-peak result at some angle but are well separated at another. Multiple angle analysis is therefore needed for a proper insight. The 90° angle is useful because of diminished effect of unpolarised light at this angle and because scattering of large particles dominates at small angles. This allows large populations to be analysed at angles smaller than 90°, while populations of small particles are better treated at larger angles.

When angular dependence is observed in R_h values, zero angle hydrodynamic radius ($R_{h,0}$) is needed for particle shape characterization through the shape parameter ρ (= $R_g/R_{h,0}$; R_g is the radius of gyration; see below). Forward light scattering measurement ($\theta = 0°$) is problematic, therefore, $R_{h,0}$ is normally determined by extrapolation of the experimental data obtained at higher angles to $\theta = 0°$. It is important to keep in mind that extrapolated values can be falsely high when analysing samples that contain more than one population [2], and overestimations can occur also due to the polydispersity of the particle's within one population [3] or due to the presence of aggregates and other large particle contaminations [4]. The deviation of R_h to higher values in our samples was very evident in unfiltered samples (see Figure 1 in the main script). To avoid overestimations, we used linear function to determine the $R_{h,0}$ of the population of large particles by considering the experimental data measured at $\theta \geq 60°$ in extrapolating them to $\theta = 0°$.

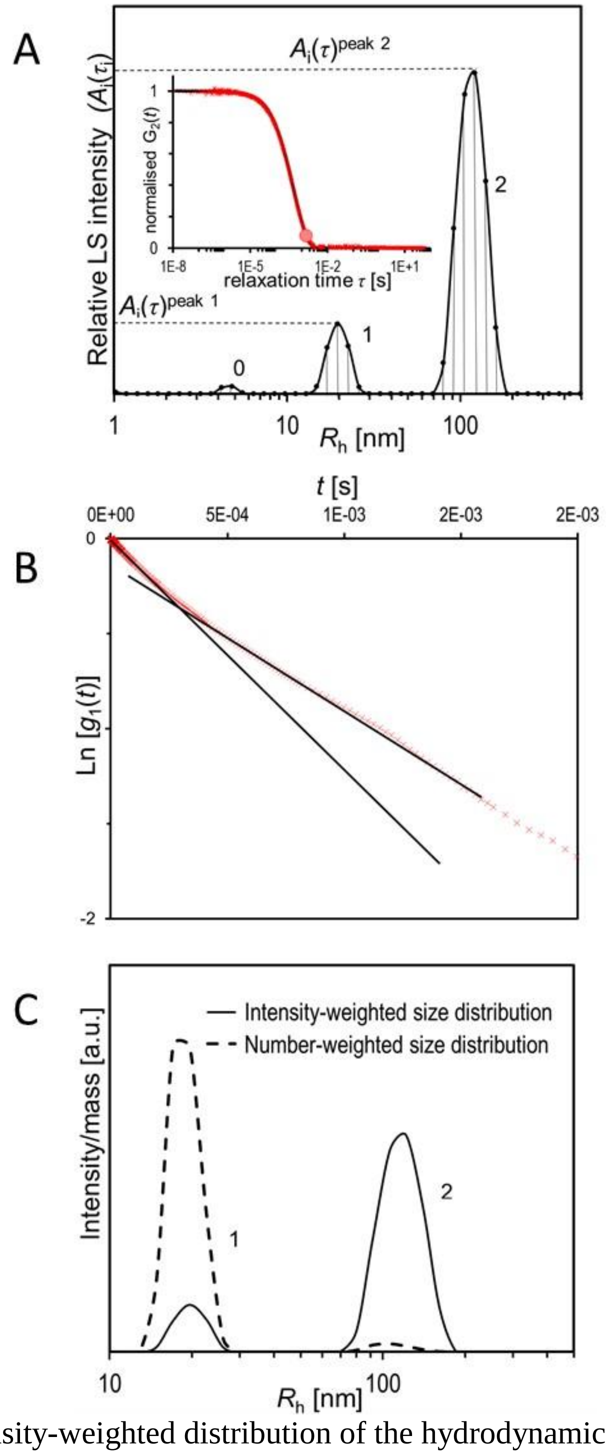

Figure S1. A) The intensity-weighted distribution of the hydrodynamic radii (R_h) obtained at $\theta = 90°$ and $T = 25°C$ for the thawed sample ES1. The Y-axis shows the relative contributions ($A_i(\tau_i)$) of particles of size $R_{h,i}$ to the total LS intensity. The measured correlation function of the intensity of scattered light, $G_2(t)$, is shown in the inset of Figure S1A; B) The $lng_1(t)$ plot for ES1 (the lines indicate slopes corresponding to populations of vesicles) ; C) intensity-, mass-, and number-weighted distributions for the same sample.

S1.1.2 Static light scattering.

The integral time averaged intensities, $I(\theta) \equiv I(q)$, were recorded in the θ-range from 30° to 150° simultaneously with the correlation functions. Intensities measured in counts of photons per second (cps) were normalized with respect to the Rayleigh ratio, R, of toluene and converted into the absolute intensity units given in cm^{-1}. The intensities of the individual populations (peaks) obtained by the below outlined procedure (see equations S4 and S5) were analysed separately for the angular dependency.

Measurements of the angular dependency of scattered light enable the determination of the form factor, $P(q)$, of colloidal particles in solution. $P(q)$ is defined as:

$$P(q) = \frac{I(q)}{I(0)} \tag{S3}$$

where $I(0)$ and $I(q)$ are the scattering intensities at θ (or q) = 0 and at an arbitrary θ (q), respectively. From $P(q)$, the radius of gyration, R_g, of particles, was obtained.

In order to calculate the R_g of the scattering particles, *i.e.* extracellular vesicles in the present study, which are represented by peaks 1 and 2 in R_h distributions (*populations 1* and *2*; the corresponding R_h designations are R_h^{peak1} and R_h^{peak2}, respectively; see manuscript and Figure S1), the total LS intensity is split into contributions, $I_1(q)$ and $I_2(q)$, resulting from these two peaks. The splitting is based on the intensity weighted distributions of the relaxation times τ. Each scattering species of the radius $R_{h,i}$ is represented in this distribution by its relative contribution, *i.e.* by the amplitude $A_i(\tau_i)$, to the total intensity of scattered light, $I_t(q)$ (see Figure S1). The relative amplitudes of the two peaks are then estimated using the procedure outlined in references [5-7] as

$$A_1 \equiv \sum_{peak1} A_i(\tau_i) \quad \text{and} \quad A_2 \equiv \sum_{peak2} A_i(\tau_i) \tag{S4}$$

and converted into the LS intensities for each population of particles. The time-averaged intensities of peaks 1 and 2, $I_1(q)$ and $I_2(q)$, respectively, are calculated as:

$I_1(q) = A_1(q) \cdot I_t(q)$ and $I_2(q) = A_2(q) \cdot I_t(q)$ (S5)

These data are then treated separately for the angular dependency. Details of this procedure can be found elsewhere [5-7].

In view of the small size of exosomes (*population 1*), the intensity of scattered light does not significantly depend on the angle. Therefore, a trustable R_g determination is not possible.

It is well-known that large particles scatter more light than the small ones and they dominate in the overall scattering, as can be seen also for our samples in Figure S1a. Intensity weighted distributions of R_h, such as in this figure, are therefore often transformed into the mass weighted distributions by taking into account that the scattering intensity is proportional to molar mass, M_i, and concentration, c_i, of a particle. M_i is further proportional to the particle radius, R_i. The following proportionality thus applies: $I_i \propto R_i^a c_i$ or $c_i \propto I_i / R_i^a$, where the exponent a depends on the particle shape (it is for example $a = 3$ for a hard sphere). Based on this relationship, the relative weight content of particles can be estimated from:

$$w_i = \frac{I_i / R_i^3}{\Sigma I_i / R_i^3} \quad \text{(S6)}$$

Figure S1 shows an example of the transformation of intensity weighted distribution (Figure S1A) into the mass weighted distribution (Figure S1B) for the same sample.

Generally, functions for R_g derivation are valid for particles that are small in comparison to the wavelength of light, *i.e.* that fulfil the criterion $qR_g < 1.5$, which was the case with particles of *population 1*. To meet the same criterion, small angle scattering is relevant for R_g determination and structural characterization for the *population 2* (larger vesicles). Either first (for $R_g \approx 25$ nm; peak 1) or second (for higher R_g, peak 2) order fit was used to fit the $P(q)$ as a function of q^2 (Eq. S3) according to equation S3a:

$$P(q) = 1 - \frac{(qR_g)^2}{3} + \left(\frac{(qR_g)^2}{3}\right)^2 \quad \text{(S3a)}$$

S1.1.3 Shape characterization through LS analysis

S1.1.3.1 Form factor of a vesicle.

The most relevant shape for a vesicle is that of a hollow sphere surrounded by a shell, as sketched in Scheme S1.

Scheme S1.

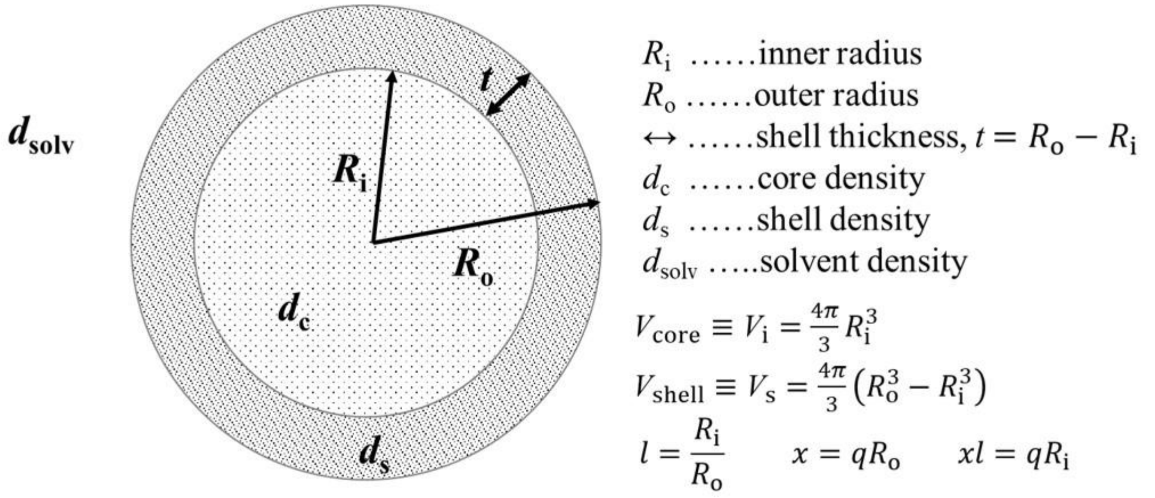

Form factor of a monodisperse spherical vesicle was derived as the difference between the form factors of 2 solid spheres with radii R_i (inner radius of the vesicle) and R_o (outer radius of the vesicle) as [8-10]:

$$P(q) = \left[\frac{3}{R_o^3 - R_i^3}\right]^2 \left[R_o^3 \frac{j_i(qR_o)}{qR_o} - R_i^3 \frac{j_i(qR_i)}{qR_i}\right]^2 \quad \text{(S3b)}$$

where $j_i(z)$ is the Bessel function of the first order.

By introducing the dimensionless parameters $l = \frac{R_i}{R_o}$ and $x = qR_o$ (giving $xl = qR_i$), the final expression for $P(q)$ gets the form [8]:

$$P(q) = \left[\frac{3}{x^3(1-l^3)}\right]^2 [sinx - xcosx - sinxl + xlcos(xl)]^2 \quad \text{(S3c)}$$

$P(q)$ is usually presented in the form of a Kratky plot, *i.e.* the dependence of $(qR_g)^2 \times P(q)$ on qR_g. This graph has an advantage that the very low scattering intensities at large scattering angles are amplified by the weight $(qR_g)^2$, making differences between various spheroidal

structures more evident. Figure S2 shows the dependence of $(qR_g)^2 \times P(q)$ on qR_g for a hard sphere, a hollow sphere with an infinitely thin shell and for a vesicle with a shell of thickness corresponding to $l = 0.75$. Characteristic oscillations in these functions are clearly seen.

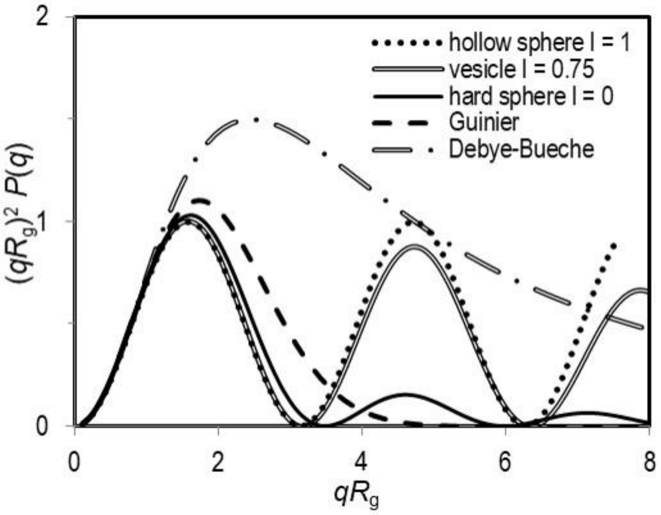

Figure S2. The Kratky plot (dependence of $(qR_g)^2P(q)$ on qR_g) for a hard sphere (l = 0), hollow sphere (l = 1) and a vesicle with l = 0.75. For comparison, Zimm, Guinier and Debye-Bueche functions are plotted as well (see text).

S1.1.3.2 Some other form factors.

For the present study, form factors of some other particle architectures appeared useful. The data for the *population 2* particles often agreed with the function (Equation S3d) suggested by Debye and Bueche [11,12], and Isihara [13], for the description of microgel-like particles with a core-shell structure having a spherical distribution of points around the centre of gravity such as in gels, networks, cross-linked or branched polymers etc., with a higher mass density in the centres:

$$P(q) = \frac{1}{\left(1 + {(qR_g)^2}/{6}\right)^2} \qquad \text{(S3d)}$$

A useful scattering function (Equation S3e) for globular structures having a spherical distribution of points around the centre of gravity with the Gaussian probability is also the Guinier function:

$$P(q) = e^{-\frac{(qR_g)^2}{3}} \quad \text{(S3e)}$$

These *P(q)* functions are also plotted in Figure S2 in the form of Kratky plots.

For polydisperse samples, the peaks for spherical shapes in the Kratky plots broaden and make the difference between hollow and solid spheres difficult to distinguish [3]. In addition, aggregation and polydispersity also affect the results. Often, the experimental data in the Kratky plot show an upward curvature at higher q (or qR_g), which is a characteristic feature of aggregation [14], and positive deviations from the theoretical function for a vesicle towards the Debye and Bueche [14,15].

S1.2 Flow cytometry (FCM)

Particle count was performed using 25 μl of samples by MACS Quant flow cytometer (Miltenyi Biotec GmbH, Bergisch-G Ladbach, Germany) with 405 nm, 488 nm, and 640 nm air-cooled lasers. Density plots of the forward scatter/side scatter light were used in the analysis. MACSQuantify (Miltenyi Biotec GmbH, Bergisch-Gladbach, Germany) software, version 2.4, was used to acquire the data and analyse the results. First we performed a routine calibration of the MACS Quant Analyzer with calibration beads (2 μm and 3 μm in size), which enabled the successful calibration of the laser settings. Then we used 460 nm beads to adjust the voltage (channels) and set the gate, which was a reference to the EVs of interest in the isolate samples. To exclude debris from the analysis, we first measured filtered buffer (PBS) and set the trigger so that the buffer events were excluded. We selected the hyper log (hlog) settings to all channels and set the trigger on SSC to 4 and shut off the secondary trigger. The final result of the measurement (Figure S3) is the total number of directly

detectable particles in the sample isolate, since we did not stain our samples nor use any antibodies. This method was shown previously to give clinically relevant results [16-18].

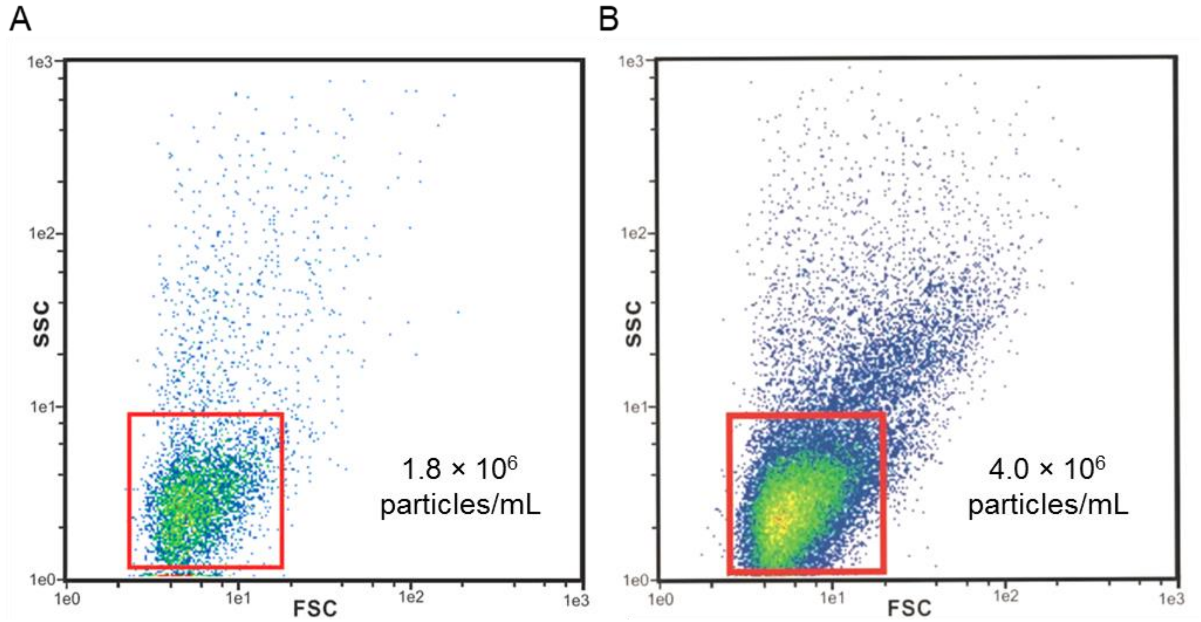

Figure S3. Gating strategy for the analysis of EVs by FCM. 2D-plot obtained by MACSQuant software for two different EV isolates, prepared from blood plasma of two healthy donors: A) HD1_EV isolate 1, B) HD2_EV isolate 1. After gating the EVs (red gate) we evaluated the total number of events in the gate (see number given in the figures). FSC – forward scatter, SSC – side scatter.

S2 Results

S2.1 Batch DLS/SLS analysis.

Figure S4 represents representative correlation functions for the examples of three different types of samples – exosome standard, blood plasma and EV isolate from the same blood plasma sample, and the calculated intensity distributions calculated from them.

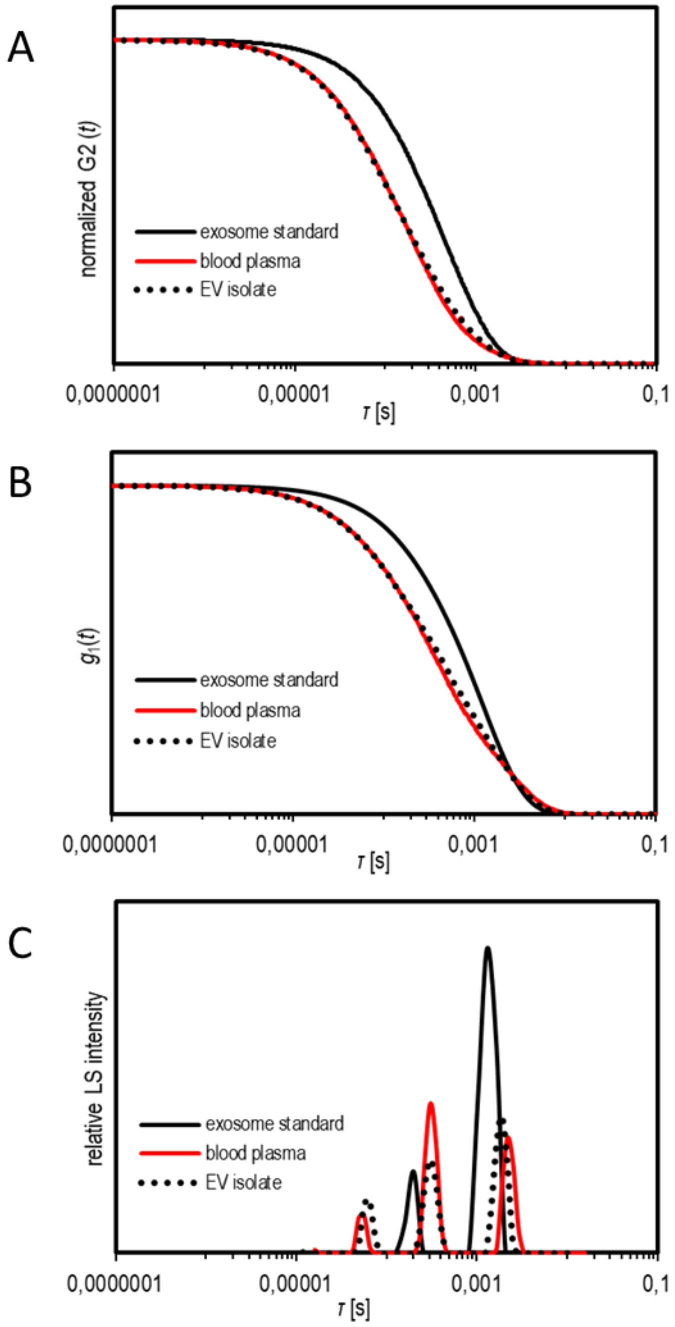

Figure S4: A) measured correlation functions ($G_2(t)$), B) calculated $g_1(t)$ and C) intensity distributions ($Ai(\tau)$) for examples of exosome standard, blood plasma and EV isolate from it.

In Tables S1-4 results of the DLS/SLS analysis of exosome standard (Table S1) and EVs in blood plasma of three healthy donors are collected.

Table S1. Results of the DLS and SLS analysis of the exosome standard: concentration of the samples is 0.1 mg/mL.

**ES**	peak	contribution to intensity [%]	$R_{h,90}$ [nm]	$R_{h,0}$ or $R_{h,aver}$* [nm]	R_g [nm]	ρ[#]
ES1	1	16	20	20*	ND	ND
fresh, unfiltered	2	84	119	162	152	0.9_4
ES1	1	10	13	14*	ND	ND
fresh, filtered	2	90	101	135	138	1.0_2
ES1	1	14	20	16*	ND	ND
thawed	2	85	118	144	160	1.1_1
ES2+TRE[a]	1	8	15	not determined		
fresh, unfiltered	2	81	114			
ES2+TRE[a]	1	17	18	16*	ND	ND
fresh, filtered	2	83	124	169	168	0.9_9
ES2+TRE[a]	1	9	11	18*	ND	ND
Thawed	2	91	113	162	164	1.0_1
ES3	1	8	16	20*	ND	ND
fresh, unfiltered	2	83	114	146	144	1.0_1
ES3	1	12	26	17*	ND	ND
fresh, filtered	2	85	104	135	135	1.0_3
ES4+TRE[a]	1	16	18	28*	ND	ND
fresh, unfiltered	2	76	139	152	156	1.0_2
ES4+TRE[a]	1	13	17	25*	ND	ND
fresh, filtered	2	86	111	146	153	1.0_4

[a] Samples ES2 and ES4 were prepared in the PBS buffer supplemented with 25 mM of trehalose (notation +TRE).

*Average R_h ($R_{h,aver}$) was calculated from the values measured at various angles when the angular dependency was not detected.

[#] The index number in ρ values denotes the unreliable second decimal number.

ND = not defined

Table S2. Results of the DLS and SLS analysis for the healthy donor 1 (HD1).

**HD1**	Peak	Contribution to intensity [%]	$R_{h,90}$ [nm]	$R_{h,0}$ or $R_{h,aver}$* [nm]	R_g [nm]	$\rho^{\#}$	$\rho^{corr\#}$
Fresh sample	1	40	21	20*	ND	ND	ND
	2	54	184	253	205	0.8_1	1.0_9
Fresh sample +TRE [a]	1	43	24	31*	47	1.5	ND
	2	49	173	259	203	0.7_8	1.0_5
Thawed, unfiltered	1	38	20	28*	48	1.7	ND
	2	58	185	207	154	0.7_4	1.0_0
Thawed, filtered	1	38	20	18*	27	1.5	ND
	2	58	185	209	150	0.7_2	0.9_7
Thawed sample +TRE [a], unfiltered	1	36	33	29*	39	1.3	ND
	2	49	177	241	202	0.8_4	1.1_3
Thawed sample +TRE[a], filtered	1	34	18	24*	43	1.8	ND
	2	57	103	199	143	0.6_1	0.8_2
HD1_EV isolate 1	1	24	15	15*	26	1.7	NC
	2	69	118	178	143	0.8_0	NC
HD1_EV isolate 2	1	25	17	21*	ND	ND	ND
	2	61	122	168	151	0.9_4	NC
HD1_EV isolate 3	1	40	17	23*	35	1.5	NC
	2	38	103	158	123	0.7_8	NC

[a] Samples were supplemented with 300 mM trehalose in the final concentration of 25 mM (notation +TRE).

* Average R_h ($R_{h,aver}$) was calculated from the values measured at various angles when the angular dependency was not detected.

[#] The index number in ρ values denotes the unreliable second decimal number. Corrected ρ values (ρ^{corr}) were calculated based on the approximation η_0 = 1.2 mPas for the plasma medium viscosity.

ND = not defined

NC = no correction. For diluted samples such as EV isolates it was assumed that dilution was sufficient to overcome the viscosity effect and η_0 = 0.9 mPas (water at 25°) was employed in evalouation of R_h. Due to the unknown value of effective viscosity of the medium for small particles, results of population 1 were never corrected.

Table S3. Results of the DLS and SLS analysis for the healthy donor 2 (HD2).

**HD2**	Peak	Contribution to intensity [%]	$R_{h,90}$ [nm]	$R_{h,0}$ or $R_{h,aver}$* [nm]	R_g [nm]	ρ#	$\rho^{corr\#}$
Fresh sample	1	51	27	30	ND	ND	ND
	2	38	195	267	212	0.8_0	1.0_8
Fresh sample +TRE [a]	1	40	18	18	28	1.5	ND
	2	54	124	226	194	0.8_6	1.1_6
Thawed. unfiltered	1	53	34	38	48	1.3	ND
	2	36	333	327	207	0.6_3	0.8_5
Thawed. filtered	1	49	38	28*	38	1.3	ND
	2	36	230	306	195	0.7_5	1.0_1
Thawed sample +TRE [a]. unfiltered	1	43	25	42	66	1.6	ND
	2	47	216	434	236	0.5_4	0.7_3
Thawed sample +TRE [a]. filtered	1	56	40	30	40	1.3	ND
	2	29	272	349	249	0.7_1	0.9_6
HD2_EV isolate 1	1	35	27	21*	29	1.4	NC
	2	49	158	174	149	0.8_5	NC
HD2_EV isolate 2	1	41	25	23*	42	1.8	NC
	2	41	145	198	155	0.7_8	NC
HD2_EV isolate 3 (thawed)	1	13	20	18*	25	1.4	NC
	2	25	126	164	112	0.6_8	NC

[a] Samples were supplemented with 300 mM trehalose in the final concentration of 25 mM (notation +TRE).

* Average R_h ($R_{h,aver}$) was calculated from the values measured at various angles when the angular dependency was not detected.

# The index number in ρ values denotes the unreliable second decimal number. Corrected ρ values (ρ^{corr}) were calculated based on the approximation η_0 = 1.2 mPas for the plasma medium viscosity.

ND = not defined

NC = no correction. For diluted samples such as EV isolates it was assumed that dilution was sufficient to overcome the viscosity effect and η_0 = 0.9 mPas (water at 25°) was employed in evalouation of R_h. Due to the unknown value of effective viscosity of the medium for small particles, results of population 1 were never corrected.

Table S4. Results of DLS/SLS analysis of healthy donor 3 (HD3).

**HD3**	Peak	Contribution to intensity [%]	$R_{h,90}$ [nm]	$R_{h,0}$ or $R_{h,aver}$* [nm]	R_g [nm]	$\rho^{\#}$	$\rho^{corr\#}$
Fresh sample	1	11	16	17*	ND	ND	ND
	2	88	174	288	166	0.5_8	0.7_8
Fresh sample +TRE [a]	1	13	16	28*	ND	ND	ND
	2	86	188	368	187	0.5_1	0.6_9
Thawed, unfiltered	1	27	41	24*	ND	ND	ND
	2	68	259	203	179	0.6_4	0.8_6
Thawed, unfiltered sample +TRE [a]	1	13	15	17*	ND	ND	ND
	2	87	189	325	193	0.5_9	0.8_0
Thawed, unfiltered	1	12	18	21*	ND	ND	ND
	2	87	191	436	224	0.5_1	0.6_9
Thawed, unfiltered, diluted 1:1 with PBS	1	10	10	18*	ND	ND	ND
	2	89	133	363	216	0.6_0	NC
Thawed, unfiltered, diluted 1:3 with PBS	1	23	20	20*	ND	ND	ND
	2	74	159	286	208	0.7_3	NC
Thawed sample +TRE [a], unfiltered	1	26	32	24*	ND	ND	ND
	2	72	242	350	218	0.6_2	0.8_4
Thawed sample +TRE [a], unfiltered, diluted 1:1 with PBS-TRE	1	15	17	18*	26	1.5	NC
	2	83	143	270	208	0.7_7	NC
Thawed sample +TRE [a], unfiltered, diluted 1:3 with PBS-TRE	1	16	18	16*	ND	ND	NC
	2	81	133	221	162	0.7_3	NC

[a] Samples were supplemented with 300 mM trehalose in the final concentration of 25 mM (notation +TRE).

* Average R_h ($R_{h,aver}$) was calculated from the values measured at various angles when the angular dependency was not detected.

[#] The index number in ρ values denotes the unreliable second decimal number. Corrected ρ values (ρ^{corr}) were calculated based on the approximation η_0 = 1.2 mPas for the plasma medium viscosity.

ND = not defined

NC = no correction. For diluted samples such as EV isolates it was assumed that dilution was sufficient to overcome the viscosity effect and η_0 = 0.9 mPas (water at 25°) was employed in evalouation of R_h. Due to the unknown value of effective viscosity of the medium for small particles, results of population 1 were never corrected.

S2.2 Effects of filtration

In sample preparation immediately before the DLS and SLS measurements, filtration and centrifugation are routinely used to eliminate large particle contaminations (for example dust particles) that can represent a substantial interference in LS due to their strong contribution to the measured LS intensity. However, the resistance of EVs to sheer stress, which they experience during filtering, is not very well known, and so the decision whether samples should be subjected to it or not is a delicate one.

In our blood plasma samples, centrifugation was used for the preparation of plasma from the blood (see Methods Section in the manuscript), which means that most of the large contaminants were removed already in the preparation step. Sometimes centrifugation may not be sufficiently efficient, and therefore filtering is routinely employed just prior to the LS measurements, in particular in case of water suspensions. To evaluate how the filtering procedure affects the analysis of vesicles, we analysed unfiltered and filtered samples. The DLS results (R_h in dependence on q^2) and the SLS results ($\Delta R = (R - R_0)^{-1}$ in dependence on q^2) for unfiltered and filtered exosome standard are shown in Figure S5.

Scattering was stronger from the unfiltered samples; the ratio between the scattered and the incident light intensity at 90° was up to 25 % higher in the unfiltered samples than in the filtered ones. This difference in intensity was evidently smaller in samples with added trehalose (see Section S2.1.2): in this case, intensity was only up to 6 % higher in the unfiltered samples. In the unfiltered samples, larger $R_{h,0}$ was generally determined for the *population 2* (see Figure S5A), if measurements at all angles were considered in the extrapolation to zero angle. In case of filtered samples, the upward curvature of R_h values at small angles was lost and similarly, the dependency of the excess LS intensity on q^2 became linear after filtration, whereas it was curved for the unfiltered sample (see Figure S5B). All

this suggests that $R_{h,0}$ in unfiltered samples is overestimated due to the large particle contaminations.

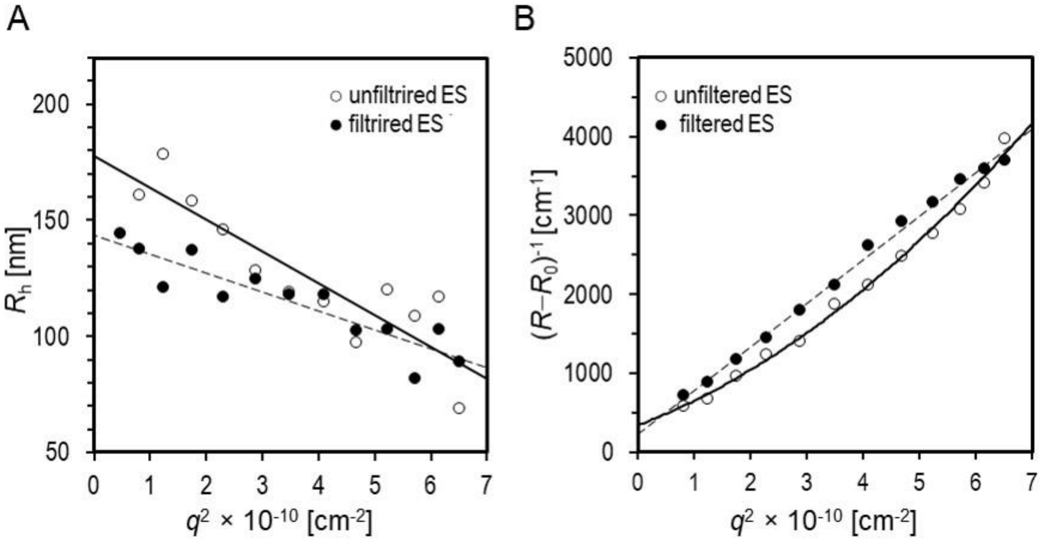

Figure S5. Comparison of the angular dependency of A) the hydrodynamic radii, R_h, and B) the reciprocal scattered light intensity, $(R - R_0)^{-1}$, measured for the *population 2* in the unfiltered (open circles) and filtered sample (full circles) of the same exosome standard (ES).

In SLS analysis, filtering usually decreased the small angle error of the measurement, but the observation that $P(q)$ turns to higher values at larger angles (larger qR_g) was present also in the filtered samples (se Figure 2 in the manuscript). The shift of $P(q)$ to higher values at larger angles is reported in the literature as well [14] and is, similarly to the present study, explained as a feature of particle aggregation.

To inspect the origin of changes in the unfiltered and filtered samples as observed by DLS/SLS, the samples were analysed also by AF4/UV-MALS and AFM. Some changes in size distributions of the population of larger particles in the filtered and unfiltered samples were confirmed by these two techniques as well (see Figures 1 and 3 in the manuscript).

In AF4/UV-MALS, the peak due to larger particles was narrower in the filtered sample. The value of R_g for larger particles in the unfiltered (filtered) sample as calculated from AF4/UV-MALS was 93 (84) nm, which is lower than the $R_{h,90}$ value for larger particles as obtained by

batch DLS ($R_{h,90}$ = 100–120 nm), and also lower than R_g calculated from SLS (R_g = 130–150 nm).

While both, batch DLS/SLS and AF4/UV-MALS showed differences between the unfiltered and filtered sample, filtration did not eliminate the discrepancy of size parameters obtained by these two techniques, suggesting a large effect of polydispersity on the batch DLS/SLS results. Polydispersity therefore may explain the change of regression curve of R_h vs. q^2 on Figure S5A, which was not shifted in parallel toward higher values in unfiltered sample, but rather it exhibited a greater slope (while the data in the θ range 80–100° remained similar to these for the filtered sample).

AFM was employed to reveal potential morphological changes of vesicles in exosome standards caused by filtration (Figures 1A and 1B in the main script). Similar spheroidal shape of particles was observed in both AFM micrographs so as the grouping of the vesicles into aggregates. Particles' size in filtered and unfiltered ES was similar, but particles in the filtered sample seemed deformed. It has to be noted, however, that the area inspected by AFM was very small and may not be representative for the whole sample. Besides, reproducible preparation of samples for AFM is difficult. The above differences may be associated with these particular experimental procedures in AFM. Finally, we concluded that AFM results confirmed our anticipations obtained from scattering techniques, *i.e.* large polydispersity and possible aggregation of vesicles. Based on results of all three techniques, degradation of vesicles upon filtration could not be excluded.

All in all, in our study, the batch DLS results for $R_{h,0}$ determination before and after filtration agreed very well if only angles larger than 50° were taken into account in the extrapolation procedure to zero angle (*c.f.* Figure S5A). Because we cannot ascertain if the decrease in the mean size and change in the form factor ($P(q)$) of larger particles in filtered samples originates from the removal of contaminations, degradation of the large vesicles into smaller

ones or dissociation of exosomes from some aggregates, we suggest that unfiltered samples can be analysed if there is a risk of sample change due to filtration. However, small angles should be excluded in the analysis of the DLS data to prevent an overestimation of $R_{h,0}$.

S2.3 Effects of freezing and trehalose addition

Freezing is a convenient and widely used storage method for biological samples such as blood plasma, but cycles of freezing and thawing may induce vesicle rifting. Fresh and frozen samples were therefore analysed by DLS/SLS to evaluate the effect of sample change due to these procedures on results of the analysis. In our study, the short-time freezing did not substantially change the size and shape of particles as demonstrated by DLS/SLS analysis, but the decrease of the scattering intensity suggests that this procedure could cause some vesicle disintegration and accompanying protein release.

The ratio between scattered and incident light intensity was consistently lower in the thawed samples of the exosome standard, and oppositely, higher in the samples of blood plasma, while the size parameters and the ρ-ratio remained similar within the limits of the experimental error (see data for the thawed samples in Tables S1–4; total LS intensity is not reported). Considering these results, the size characterization after one cycle of freezing and thawing was taken as still reliable. The decrease in light intensity ratio in samples of thawed exosome standards was attributed to some rifting of vesicles, whereas its increase in thawed blood plasma samples may be due to the aggregation of vesicles and/or proteins.

Parallel samples were prepared with trehalose to assess its potential benefit for cryo-protection and aggregation prevention. In the results reported in Tables S1–4, the differences between samples with and without trehalose were not significant enough to prove its function. It is possible that a higher concentration of trehalose would have a different effect.

S2.2 Ultracentrifugation experiments

S2.2.1 Preliminary sedimentation test

Based on the literature reports [19-22] it was predicted that after 15 min centrifugation at 10 000 × g at 4 °C particles larger of 200 nm will accumulate in the pellet and after 1 h at 100 000 × g the vesicles of 30 nm would be precipitated too. Plasma sample was centrifuged in sever cycles to monitor particle sedimentation. The fractions obtained in experiment (Exp1) are described below.

Exp1. s0: supernatant after *15 min at 1500 × g, 22 °C.*

Exp1. sI (aliquots no.1 and 2):

Supernatant I: Supernatant of two aliquots (volume: 2 × 1.25 mL) after 15 min centrifugation at 10 000 × g. Pellet was discarded due to visual presence of cells.

Exp1. sII (aliquots no. 3 and 4):

Supernatant II: Two supernatant of first centrifugation (volume: 2 × 1400 µL) were transferred into fresh tubes and centrifuged again for 1 h at 100 000 × g (38 000 RPM). Supernatants (final volume 2 × 1.15 µL) were pooled for the viscosity, density and DLS measurements.

Pellet II: 2× 250 µL of the sample (pellet + remaining plasma) left in the tube after the second centrifugation was resuspended in 800 µL of PBS-citrate buffer.

Exp1. sIII (aliquots n. 5 and 6):

Supernatant III: Two supernatant of the first centrifugation (volume: 2 × 1400 µL) were transferred into fresh tubes and centrifuged again for 1 h at 100 000 × g (38 000 RPM). Supernatants (final volume 2 × 1300 µL) were transferred into fresh tubes, centrifuged again for 1 h at 100 000 × g (38 000 RPM). Supernatants (2 × 1100 µL) were pooled for the viscosity, density and DLS measurements.

Pellet III: 2 × 200 µL of the sample (pellet + remaining plasma) left in the tube after the last cycle was resuspended in 800 µL of PBS buffer.

Exp1. sIV (aliquots n. 7 and 8):

Supernatant IV: Two supernatant of first centrifugation (2 × 1400 µL) of first centrifugation (volume: 2 × 1400 µL) were transferred into fresh tubes, and centrifuged for three 1-h cycles at 100 000 × g (38 000 RPM). Supernatants (final volume 2 × 1250 µL) were pooled for the measurements of viscosity, density and DLS.

Pellet IV: 2 × 150 µL of the sample (pellet + remaining plasma) left in the tube after supernatant removal was resuspended in 800 µL of PBS buffer.

Exp1. sV – EV isolate (aliquots 5-I and 6-I): 1.2 mL of PBS were added to pellets of Exp1. sIII (2 × 100 µL) after the first ultracentrifugation (1 h at 100 000 × g), resuspended and centrifuged again for 1 h at 100 000 × g. The supernatants (2 × 1.2 mL) were discarded. 1.2 mL PBS was added to remaining pellets. Samples were resuspended and centrifuged again for 1 h at 100 000 × g. 1 mL of supernatants were discarded, while 0.4 mL PBS was added to the remaining 0.2 mL of pellet in each tube and resuspended.

Exp1.s0 and the supernatants I-IV were measured for their density and viscosity. The results are given in the Table S5.

Table S5. Density (*d*) and dynamic viscosity (η) of fractions obtained in the preliminary sedimentation test EXP1 at 25°C (see above).

	d [g/cm^3]	η [mPas]
Water	0.997	0.90
Exp1.s0	1.024	1.46
Supernatant I	1.024	1.46
Supernatant II	1.024	1.45
Supernatant III	1.024	1.44
Supernatant IV	1.024	1.43

The samples were analysed by DLS (results in Table S6). All the methods suggest that the final separation of the sample was hardly changed even after 4 cycles of ultracentrifugation at 100 000 × g.

Table S6. Results of DLS analysis for fractions obtained in the preliminary EV sedimentation test Exp1.

	peak	contribution to intensity [%]	$R_{h,90}$ [nm]
Exp1.s0	1	1	2
	2	11	7
I (total) = 765 kHz/mW	3	77	50
	4	12	328
Supernatant I	1	5	4
	2	8	10
I (total) = 815 kHz/mW	3	70	42
	4	17	313
Supernatant II	1	7	3
I (total) = 495 kHz/mW	2	46	17
	3	48	66
Pellet II	1	4	3
I (total) = 281 kHz/mW	2	40	16
	3	55	54
Supernatant III	1	9	4
I (total) = 425 kHz/mW	2	31	13
	3	60	54
Pellet III	1	2	3
I (total) = 533 kHz/mW	2	26	14
	3	73	54
Supernatant IV	1	6	3
I (total) = 467 kHz/mW	2	43	16
	3	51	67
Pellet IV	1	3	3
I (total) = 452 kHz/mW	2	34	15
	3	64	61
EV isolate	1	2	5
I (total) = 28 kHz/mW	2	25	27
	3	65	133

S2.2.2 Plasma fractions obtained after 8h of ultracentrifugation at 100 000 × g

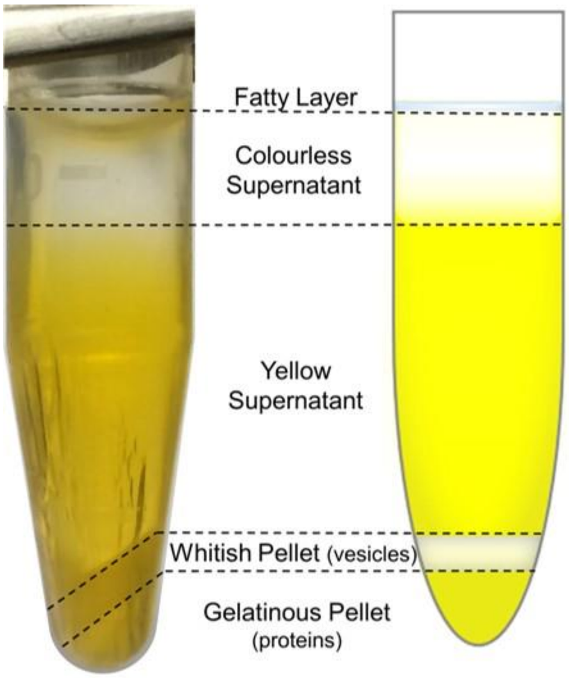

Figure S6: After 8 h of ultracentrifugation at 100 000 × g, blood plasma separates into five distinguishable layers.

Table S7. Dynamic viscosity (η) of various samples at 25 °C.

Sample	d [g/cm^3]	η [mPas]
Water	0.997	0.90
Blood plasma (initial sample)	1.024	1.47
Colourless supernatant (*c.f.* Figure S6)	1.008	0.94
Yellow supernatant (*c.f.* Figure S6)	1.021	1.32
Total supernatant (colourless s. + yellow s.)	1.019	1.21

S2.2.3 Determination of protein content

Table S8. Estimation of total protein contents in plasma fractions obtained after 8-h of ultracentrifugation at 100 000 × g.

	A_{260}/A_{280}	Total Proteins [g/L]
Initial plasma sample	0.61	39
Colourless supernatant	0.73	4.2
Colourless supernatant of PBS diluted sample 1:1	0.70	2.7
Colourless supernatant of PBS diluted sample 1:2	0.70	1.6
Yellow supernatant	0.61	43
Yellow supernatant of PBS diluted sample 1:1	0.60	29
Yellow supernatant of PBS diluted sample 1:1	0.59	22
Complete pellet suspended in the yellow supernatant	0.59	90
Complete pellet	0.57	159
Gel pellet	0.55	153

Measurements were performed at the Faculty of Chemistry and Chemical technology. University of Ljubljana on the spectrometer NanoDrop, Thermo Scientific, USA. Concentrations were calculated based on equation: [proteins] mg/mL = 1.55 × A_{280} – 0.76 × A_{260}; and adjusted for dilution factor if fraction was diluted upon preparation for analysis.

S2.2.4 SDS-PAGE determination of protein content

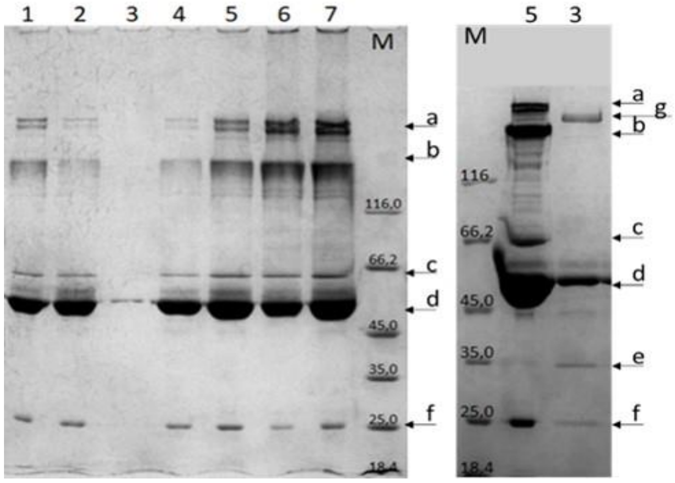

Figure S7: SDS-PAGE of blood plasma and its fractions obtained after 8-h ultracentrifugation (100 000 × g). Samples were loaded on 10 % in amount corresponding 0.1 μL of undiluted fraction (for the left gel) and 7.5 μL and 15 μL of samples 5 and 3, respectively (on the right gel).

1 – Initial plasma sample (final cell debris removed by 15 min centrifugation at 1500 × g one day after blood donation)
2 – Initial plasma sample (final cell debris removed by 30 min centrifugation at 5000 × g three days after blood donation)
3 – Colourless supernatant
4 – Yellow supernatant
5 – Pellet in yellow supernatant
6 – Pellet in yellow supernatant (sample was diluted with pbs 1:1 before ultracentrifugation)
7 – Gel pellet
M – Size marker (size in kDa is annotated on the picture)
Typical plasma proteins: a – fibrinogen. b – IgG. c – transferrin. d – albumin. e – apoE. f – light chains of IgG, apoA1. g – apoB. Bands were assigned according to their mass and comparison of their electrophoretic mobility described in literature.

S4 References

1. Provencher, S.W. CONTIN: A General Purpose Constrained Regularization Program for Inverting Noisy Linear Algebraic and Integral Equations. *Computer Physics Communications* 1982, pp 229-242, doi:10.1016/0010-4655(82)90174-6.
2. Naiim, M.; Boualem, A.; Ferre, C.; Jabloun, M.; Jalocha, A.; Ravier, P. Multiangle dynamic light scattering for the improvement of multimodal particle size distribution measurements. *Soft Matter* **2015**, *11*, 28-32, doi:10.1039/C4SM01995D.
3. Kaasalainen, M.; Aseyev, V.; von Haartman, E.; Karaman, D.S.; Makila, E.; Tenhu, H.; Rosenholm, J.; Salonen, J. Size, Stability, and Porosity of Mesoporous Nanoparticles Characterized with Light Scattering. *Nanoscale Res Lett* **2017**, *12*, 74, doi:10.1186/s11671-017-1853-y.
4. Hassan, P.A.; Rana, S.; Verma, G. Making Sense of Brownian Motion: Colloid Characterization by Dynamic Light Scattering. *Langmuir* **2015**, *31*, 3-12, doi:10.1021/la501789z.
5. Klucker, R.; Munch, J.P.; Schosseler, F. Combined Static and Dynamic Light Scattering Study of Associating Random Block Copolymers in Solution. *Macromolecules* **1997**, *30*, 3839-3848, doi:10.1021/ma961710l.
6. Raspaud, E.; Lairez, D.; Adam, M.; Carton, J.P. Triblock Copolymers in a Selective Solvent. 1. Aggregation Process in Dilute Solution. *Macromolecules* **1994**, *27*, 2956-2964, doi:10.1021/ma00089a011.
7. Tarassova, E.; Aseyev, V.; Filippov, A.; Tenhu, H. Structure of poly(vinyl pyrrolidone) – C70 complexes in aqueous solutions. *Polymer* **2007**, *48*, 4503-4510, doi:10.1016/j.polymer.2007.05.069.
8. Pecora, R.; Aragón, S.R. Theory of light scattering from hollow spheres. *Chemistry and Physics of Lipids* **1974**, *13*, 1-10, doi:10.1016/0009-3084(74)90037-1.
9. Pencer, J.; Hallett, F.R. Effects of Vesicle Size and Shape on Static and Dynamic Light Scattering Measurements. *Langmuir* **2003**, *19*, 7488-7497, doi:10.1021/la0345439.
10. Schärtl, W. *Light Scattering from Polymer Solutions and Nanoparticle Dispersions - Springer*; Springer Laboratory: 2007. doi:10.1007/978-3-540-71951-9.
11. Debye, P.; Bueche, A.M. Scattering by an Inhomogeneous Solid. *Journal of Applied Physics* **1949**, *20*, 518-525, doi:10.1063/1.1698419.
12. Debye, P.; Bueche, F. Distribution of Segments in a Coiling Polymer Molecule. *The Journal of Chemical Physics* **1952**, *20*, 1337-1338, doi:10.1063/1.1700751.
13. Isihara, A. Behavior of linear polymers in solution. *Journal of Polymer Science* **1952**, *8*, 574-576, doi:10.1002/pol.1952.120080515.
14. Savin, G.; Burchard, W. Uncommon Solution Behavior of Poly(N-vinylimidazole). Angular Dependence of Scattered Light from Aggregates in Ethanol. *Macromolecules* **2004**, *37*, 3005-3017, doi:10.1021/ma0353639.
15. Stauch, O.; Schubert, R.; Savin, G.; Burchard, W. Structure of Artificial Cytoskeleton Containing Liposomes in Aqueous Solution Studied by Static and Dynamic Light Scattering. *Biomacromolecules* **2002**, *3*, 565-578, doi:10.1021/bm0200074.
16. Mrvar-Brecko, A.; Sustar, V.; Jansa, V.; Stukelj, R.; Jansa, R.; Mujagic, E.; Kruljc, P.; Iglic, A.; Haegerstrand, H.; Kralj-Iglic, V. Isolated microparticles from peripheral blood and body fluids as observed by scanning electron microscope. *Blood Cells Mol Dis* **2010**, *44*, 307-312, doi:10.1016/j.bcmd.2010.02.003.
17. Štukelj, R.; Schara, K.; Bedina - Zavec, A.; Šuštar, V.; Pajnič, M.; Pađen, L.; Krek, J.L.; Kralj-Iglič, V.; Mrvar-Brečko, A.; Janša, R. Effect of shear stress in the flow through the sampling needle on concentration of nanovesicles isolated from blood. *European Journal of Pharmaceutical Sciences* **2017**, *98*, 17-29, doi:10.1016/j.ejps.2016.10.007.
18. Šuštar, V.; Bedina-Zavec, A.; Štukelj, R.; Frank, M.; Bobojević, G.; Janša, R.; Ogorevc, E.; Kruljc, P.; Mam, K.; Šimunič, B., et al. Nanoparticles isolated from blood: a reflection of vesiculability of blood cells during the isolation process. *International Journal of Nanomedicine* **2011**, *6*, 2737-2748, doi:10.2147/IJN.S24537.

19. Menck, K.; Bleckmann, A.; Schulz, M.; Ries, L.; Binder, C. Isolation and Characterization of Microvesicles from Peripheral Blood. *J Vis Exp* **2017**, doi:10.3791/55057.
20. Li, P.; Kaslan, M.; Lee, S.H.; Yao, J.; Gao, Z. Progress in Exosome Isolation Techniques. *Theranostics* **2017**, *7*, 789-804, doi:10.7150/thno.18133.
21. Livshits, M.A.; Khomyakova, E.; Evtushenko, E.G.; Lazarev, V.N.; Kulemin, N.A.; Semina, S.E.; Generozov, E.V.; Govorun, V.M. Isolation of exosomes by differential centrifugation: Theoretical analysis of a commonly used protocol. *Scientific Reports* **2015**, *5*, 17319, doi:10.1038/srep17319.
22. Gould, S.J.; Raposo, G. As we wait: coping with an imperfect nomenclature for extracellular vesicles. *J Extracell Vesicles*, 2013; *2*, 2, doi:10.3402/jev.v2i0.20389.
